# PTHrP intracrine actions divergently influence breast cancer growth through p27 and LIFR

**DOI:** 10.1186/s13058-024-01791-z

**Published:** 2024-02-26

**Authors:** Courtney M. Edwards, Jeremy F. Kane, Jailyn A. Smith, Déja M. Grant, Jasmine A. Johnson, Maria A. Hernandez Diaz, Lawrence A. Vecchi, Kai M. Bracey, Tolu N. Omokehinde, Joseph R. Fontana, Breelyn A. Karno, Halee T. Scott, Carolina J. Vogel, Jonathan W. Lowery, T. John Martin, Rachelle W. Johnson

**Affiliations:** 1 Graduate Program in Cancer Biology, Vanderbilt University, Nashville, TN USA; 2 Vanderbilt Center for Bone Biology, Vanderbilt University Medical Center, Nashville, TN USA; 3 Department of Medicine, Vanderbilt University Medical Center, Nashville, TN USA; 4 Meharry Medical College, Nashville, TN USA; 5 Department of Cell and Developmental Biology and Program in Developmental Biology, Vanderbilt University, Nashville, TN USA; 6 Vanderbilt University, Nashville, TN 37232 USA; 7 Marian University College of Osteopathic Medicine, Indianapolis, IN USA; 8 Bone and Muscle Research Group, Marian University, Indianapolis, IN USA; 9 Academic Affairs, Marian University, Indianapolis, IN USA; 10 Indiana Center for Musculoskeletal Health, Indiana University School of Medicine, Indianapolis, IN USA; 11 Bone Cell Biology and Disease Unit, St. Vincent’s Institute of Medical Research, Fitzroy, VIC Australia; 12 Department of Medicine, The University of Melbourne, St. Vincent’s Hospital, Fitzroy, VIC Australia

## Abstract

The role of parathyroid hormone (PTH)-related protein (PTHrP) in breast cancer remains controversial, with reports of PTHrP inhibiting or promoting primary tumor growth in preclinical studies. Here, we provide insight into these conflicting findings by assessing the role of specific biological domains of PTHrP in tumor progression through stable expression of PTHrP (-36-139aa) or truncated forms with deletion of the nuclear localization sequence (NLS) alone or in combination with the C-terminus. Although the full-length PTHrP molecule (-36-139aa) did not alter tumorigenesis, PTHrP lacking the NLS alone accelerated primary tumor growth by downregulating p27, while PTHrP lacking the NLS and C-terminus repressed tumor growth through p27 induction driven by the tumor suppressor leukemia inhibitory factor receptor (LIFR). Induction of p27 by PTHrP lacking the NLS and C-terminus persisted in bone disseminated cells, but did not prevent metastatic outgrowth, in contrast to the primary tumor site. These data suggest that the PTHrP NLS functions as a tumor suppressor, while the PTHrP C-terminus may act as an oncogenic switch to promote tumor progression through differential regulation of p27 signaling.

**Supplementary Information:**

The online version contains supplementary material available at 10.1186/s13058-024-01791-z.

## Introduction

Parathyroid hormone-related protein (PTHrP) is a pleiotropic hormone encoded by the *PTHLH* gene located on chromosome 12, with nine exons and at least three identified promoters [1]. In humans, alternative splicing gives rise to three mature isoforms containing 139, 141, or 173 amino acids, and the first 111 amino acids of the PTHrP sequence are highly conserved among different mammalian species [2]. Regulation of PTHrP is complex and tissue-specific, with the molecule containing numerous cleavage sites and post-translational modifications [1]. The PTHrP polypeptide contains an intracellular trafficking and secretion signal, a domain that controls binding to and activation of the classical parathyroid hormone type 1 receptor (PTH1R), and a mid-molecule domain that regulates placental calcium transport. Additionally, the molecule possesses a domain historically termed the nuclear localization sequence (NLS) from amino acids 67–94 which regulates nuclear import based on studies carried out in chondrocytes [3], and a carboxy-terminal (C-terminal) domain (beginning at residue 107), to which a number of biological activities have been ascribed [4, 5].

Beyond its well-characterized endocrine and paracrine roles in inducing hypercalcemia of malignancy [6, 7] and tumor-induced bone disease [8–11], PTHrP regulates the growth of numerous tissues through its intracrine (intracellular) effects on cell survival, proliferation, apoptosis, invasion, and migration, which can occur independent of PTHrP:PTH1R binding on the cell surface [12–15]. PTHrP acting through its classical NLS (67-94aa) alters proliferation in peripheral tissues including vascular smooth muscle [16–18], where PTHrP also has a smooth muscle relaxing effect [19, 20].

Though less well studied, PTHrP also plays an important role in tumor development. In patients, PTHrP is detectable in most primary breast tumors [11] and serum PTHrP levels are elevated in the majority of patients with hypercalcemia due to breast cancer bone metastases [21, 22]. However, studies have not identified a direct association between elevated serum PTHrP levels in patients and enhanced primary breast tumor growth. The role of PTHrP in primary breast cancer progression remains highly controversial. Some clinical studies demonstrate that PTHrP expression in the primary tumor correlates with improved patient survival and formation of fewer bone metastases [23, 24], while others report that PTHrP is associated with worse patient outcomes [11, 25, 26]. Conflicting data from pre-clinical studies have further confounded the field; genetically similar mouse models that spontaneously form mammary carcinomas have produced directly conflicting results suggesting that PTHrP can inhibit [27] or promote breast tumorigenesis [28]. Thus, the prognostic role for PTHrP in primary breast tumor progression remains largely unclear.

In contrast to its uncertain role in the primary tumor, PTHrP has a well-defined deleterious effect on patient outcomes in later stages of disease progression, where its expression drives bone colonization and metastatic tumor growth [11, 26, 29]. Bone disseminated breast cancer cells secrete osteolytic factors like PTHrP, which induces receptor activator of nuclear factor-κB ligand (RANKL)-dependent osteoclastogenesis through PTH1R activation on osteoblasts [30]. In human MCF7 breast cancer cells, which normally lie dormant in bone [9, 31–33], overexpression of PTHrP (1-139aa) reprograms the cells to become highly osteolytic and dramatically increases bone tumor burden in vivo [9]. Our studies suggest that this potentially occurs through PTHrP-mediated suppression of the breast tumor suppressor leukemia inhibitory factor receptor (*LIFR*) [32, 34, 35] and other pro-dormancy factors [30, 33–36]. Our group, and others, have reported evidence that PTHrP can regulate breast tumor progression independent of paracrine or autocrine activation of PTH1R or downstream canonical cAMP signaling [37, 38]. This suggests that PTHrP acts in an intracrine manner to influence breast tumor cell behavior. In support of this, PTHrP (38-94aa) containing the calcium transport region and NLS has been shown to bind to chromatin [39], and full-length secreted PTHrP (-36-139aa) has been shown to localize to the *LIFR* proximal promoter [40].

In this study, we sought to determine how the intracrine activity of the PTHrP NLS (67-94aa) regulates breast tumor growth and how this effect may be co-regulated by the C-terminal region, since a role for these domains had not been examined in breast cancer cells. In vitro expression of endogenous PTHrP is quite low [32] and there are no reliable antibodies to detect its endogenous isoforms or biological domains. Thus, we rely on an engineered system of expressing truncated mutant proteins with deletion of the PTHrP NLS and C-terminal domains. Our findings begin to provide insight into some of the conflicting preclinical data in the literature, which may provide a framework for targeting PTHrP and its downstream signaling mediators in breast cancer.

## Results

### Human breast cancer cells generated to express full-length PTHrP or truncated peptides

**Fig. 1:**
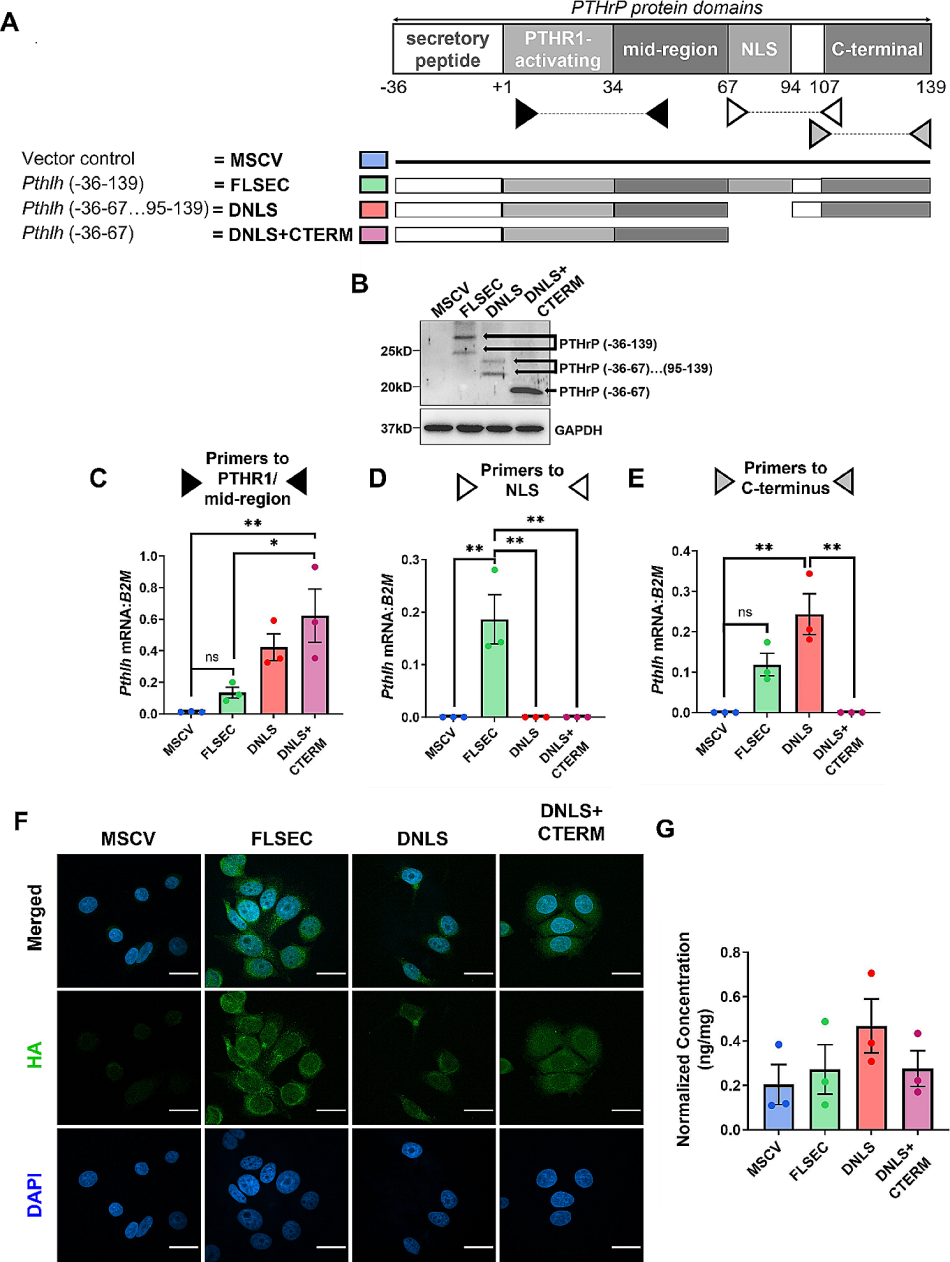
Validation of plasmids expressing specific PTHrP domains. (**A**) *Pthlh* overexpression construct design and validation in MCF7 cells by (**B**) western blot for the C-terminal HA-Tag and qPCR for the (**C**) mid-region, (**D**) nuclear localization sequence (NLS), and (**E**) C-terminal domain. MSCV = control, FLSEC = full-length secreted, DNLS = NLS deleted, DNLS + CTERM = NLS and C-terminal domain deleted. Predicted molecular weights: FLSEC PTHrP (-36-139aa) = 21.2kD, DNLS PTHrP (-36-67aa)…(95-139aa) = 18kD, DNLS + CTERM PTHrP (-36-67aa) = 12.8kD. GAPDH = loading control. (**F**) Immunocytochemical staining for HA-Tag (green) and DAPI (blue). All panels = 100X and scale bars = 25 *μ*m. (**G**) Secreted PTHrP (1-34aa) levels measured by ELISA from conditioned media of cells described in (A). (B-E & G) *n* = 3 independent biological replicates. Graphs represent mean ± SEM. (**C**) **p < 0.001 vs. MSCV or *p < 0.05 vs. FLSEC by one-way ANOVA with multiple comparisons. (**D**) **p < 0.001 vs. FLSEC by one-way ANOVA with multiple comparisons. (**E**) **p < 0.001 vs. DNLS by one-way ANOVA with multiple comparisons

To determine how PTHrP and its biological domains regulate breast tumor progression, we generated MCF7 human breast cancer cell lines that stably express different domains of the PTHrP molecule (collectively referred to herein as PTHrP mutant cell lines). The plasmids express full-length secreted PTHrP (termed FLSEC, -36-139aa), or truncated forms lacking the classical NLS alone (termed DNLS, -36-67…95-139aa) or NLS and C-terminal domain (termed DNLS + CTERM, -36-67aa) with a C-terminal HA tag that is absent in the MSCV control (Fig. 1A). We were unable to generate a mutant with deletion of the secretion signal since these cells do not survive *in vitro.* We validated plasmid expression at the protein level using an anti-HA antibody and at the mRNA level with qPCR primers targeted to amplify different regions of the *Pthlh* gene (Fig. 1B-E).

To verify expression of the plasmids and characterize the intracellular localization of the PTHrP peptides, we performed immunocytochemical staining for the C-terminal HA tag (Fig. 1F). We confirmed an absence of HA expression and fluorescence staining in the MSCV control cells as these plasmids do not contain a C-terminal HA tag. Full-length secreted PTHrP localized to both the nucleus and cytoplasm. Deletion of the NLS alone or NLS and C-terminal domain did not preclude nuclear entry as each PTHrP mutant protein was present in the nucleus as well as cytoplasm (Fig. 1F & Supplementary Fig. 1), regardless of whether they expressed the classical NLS. Therefore, these truncated PTHrP peptides likely gained entry into the nucleus independent of this recognized NLS. While we cannot modulate relative amounts of the PTHrP peptides as it is not possible to accurately engineer our model system in this manner, we observed no statistically significant difference in PTHrP levels secreted by the PTHrP mutant cell lines compared to controls as measured by an enzyme-linked immunosorbent assay (ELISA) for PTHrP (1-34aa) (Fig. 1G). Thus, altering expression of the NLS or the C-terminal domain does not affect PTHrP secretion by MCF7 cells. Additionally, differences in phenotypes between the PTHrP mutant cells are likely not due to paracrine effects of secreted PTHrP since we and others have previously shown that PTHrP does not activate PTH1R or downstream cAMP signaling in breast cancer cells [37, 38].

### The PTHrP NLS and C-terminal domain oppositely regulate breast tumor progression

**Fig. 2:**
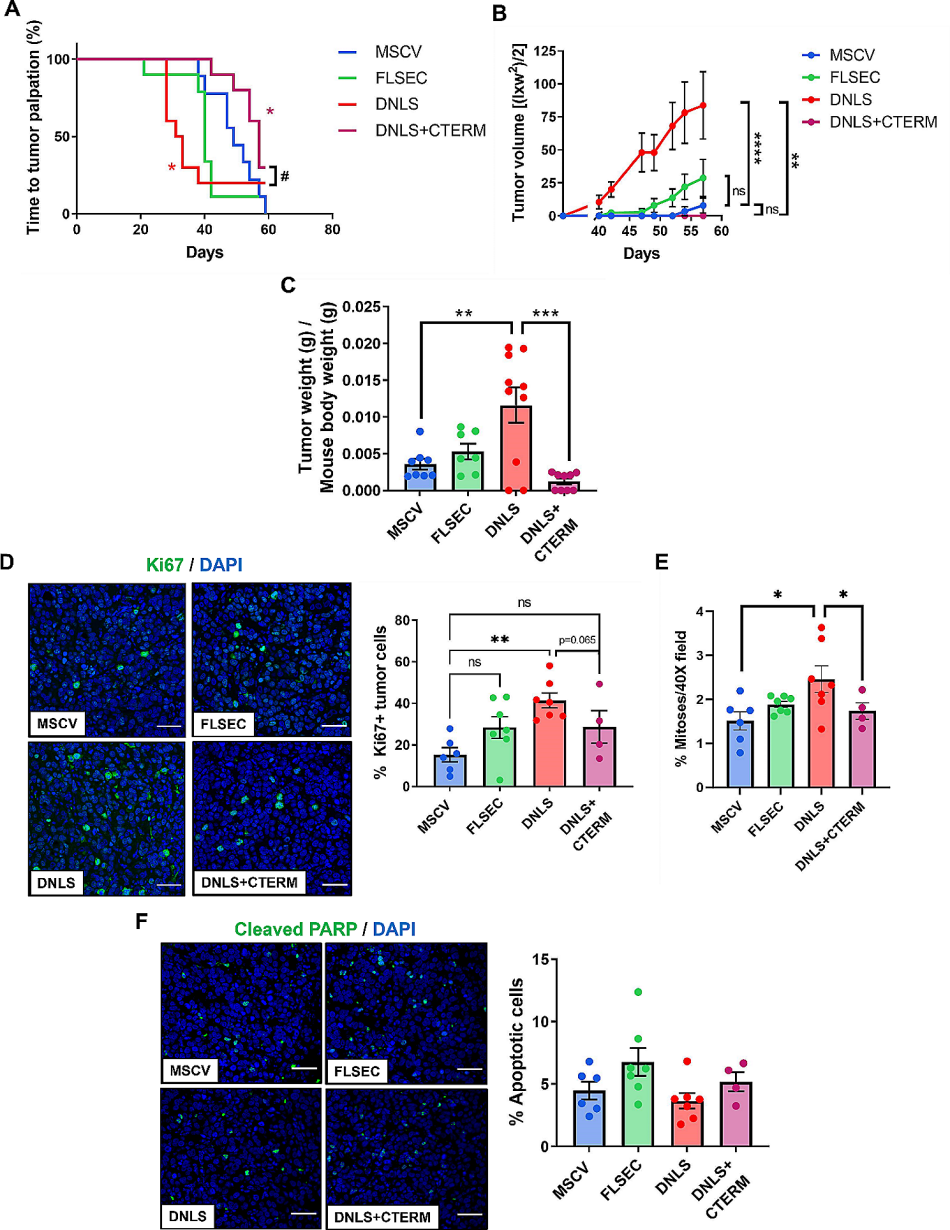
Deletion of the PTHrP NLS alters breast cancer cell proliferation and primary tumor growth. (**A**) Time to tumor palpation, (**B**) tumor volume over time by digital caliper measurement and (**C**) final tumor weight in mice inoculated with MSCV, FLSEC, DNLS, or DNLS + CTERM cells into the mammary fat pad. *n* = 7–10 mice/group. (**D**) Ki67 staining and quantification from tumors in (**A-C**). (**E**) Quantification of mitoses (# mitotic figures/total cells in 40X field) by DAPI staining from tumors in (**A-C**). (**F**) Cleaved PARP staining and quantification from tumors in (**A-C**). All panels = 40X and scale bar = 50 *μ*m. (**A**) *p < 0.05 vs. MSCV by one-way ANOVA with multiple comparisons or #*p* < 0.05 DNLS vs. DNLS + CTERM by unpaired t-test. (**B**) ****p < 0.0001 vs. MSCV by one-way ANOVA with multiple comparisons or **p < 0.01 vs. DNLS by unpaired t-test. (**C**) **p < 0.01 vs. MSCV by one-way ANOVA with multiple comparisons or ***p < 0.001 vs. DNLS by unpaired t-test. (**D**) **p < 0.01 vs. MSCV by one-way ANOVA with multiple comparisons. (**E**) *p < 0.05 vs. MSCV by one-way ANOVA with multiple comparisons or *p < 0.05 vs. DNLS by unpaired t-test. Graphs represent mean ± SEM

Next, we sought to determine how PTHrP and its biological domains regulate primary breast tumor growth in vivo. Overexpression of full-length PTHrP (-36-139aa) did not significantly alter time to tumor palpation or tumor size compared with controls (Fig. 2A-C and A: *p* = 0.0497 Log-rank, *p* = 0.0012 Gehan-Breslow-Wilcoxen; 2B: ANOVA *p* < 0.0001). Strikingly, deletion of the PTHrP NLS alone resulted in tumors that formed significantly earlier and grew larger than controls, while deletion of both the NLS and C-terminal domains completely reversed this phenotype such that the tumors grew significantly slower and smaller (Fig. 2A-C). To confirm that the PTHrP mutant plasmids were still expressed in vivo, we performed immunofluorescence staining of the primary tumors for the C-terminal HA tag, which was appropriately present in all tumors except the MSCV group, since the MSCV control plasmid does not contain an HA tag (Supplementary Fig. 2). We next assessed whether the changes in tumor size were due to increased proliferation, or reduced cell death. Deletion of the PTHrP NLS alone significantly increased the percentage of Ki67 + positive cells (Fig. 2D) and mitoses (Fig. 2E) in the primary tumors while deletion of both the NLS and C-terminal domain resulted in significantly decreased mitoses (Fig. 2E). There was no difference in cleaved PARP staining in any of the PTHrP mutant cell lines compared to MSCV controls (Fig. 2F). Collectively, these data suggest that the PTHrP NLS regulates breast tumor growth by increasing tumor cell proliferation without impacting apoptosis, but this function is abolished when the PTHrP C-terminus is deleted.

### p27 is differentially regulated by the PTHrP NLS and C-terminal domains in breast cancer

**Fig. 3:**
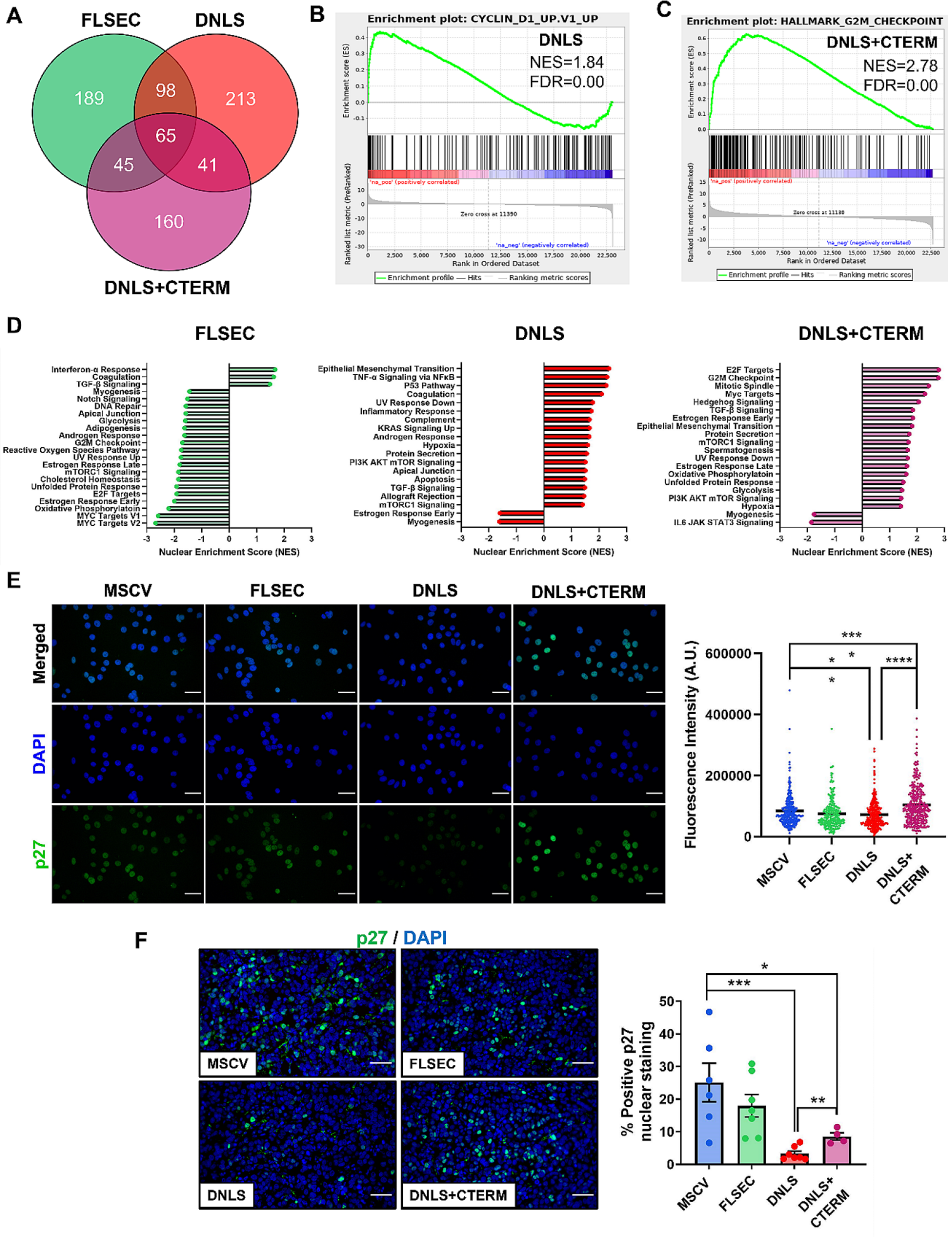
PTHrP lacking the NLS and C-terminal domain regulates proliferation by altering expression of p27. (**A**) Number of genes identified by RNAseq with log_2_fold change > 1 and *p* < 0.05. (**B**) GSEA plot from DNLS cells showing enrichment of Cyclin D1 gene signature in MCF7 cells. (**C**) GSEA plot from DNLS + CTERM cells showing enrichment of genes that regulate the G2M checkpoint. (**D**) Top twenty enriched Hallmark pathways from FLSEC, DNLS, and DNLS + CTERM cells. (**E**) Immunocytochemical staining and quantification of p27 in MSCV, FLSEC, DNLS, or DNLS + CTERM cells. *n* = 3 independent biological replicates. All panels = 40X, scale bar = 25 *μ*m. (**F**) Immunofluorescence staining and quantification for p27 in primary tumors from mice inoculated with MSCV, FLSEC, DNLS, or DNLS + CTERM cells. All panels = 40X, scale bar = 50 *μ*m. (**E**) **p < 0.01 or ****p < 0.0001 vs. MSCV by one-way ANOVA with multiple comparisons or ****p < 0.0001 vs. DNLS by unpaired t-test. (**F**) *p < 0.05 or ***p < 0.001 vs. MSCV by one-way ANOVA with multiple comparisons or **p < 0.01 vs. DNLS by unpaired t-test. Graphs represent mean ± SEM

To better understand the in vivo phenotype and mechanism by which the PTHrP NLS and C-terminal domains differentially regulate breast cancer cell proliferation, we performed RNA sequencing on the PTHrP mutant cell lines. We identified several hundred significantly altered genes (≥ log_2_ fold change 1 or ≤ log_2_ fold change − 1, *p* < 0.05) that were differentially expressed across the PTHrP mutants (Fig. 3A, Supplementary Data 1). Gene Set Enrichment Analysis (GSEA) of these data revealed that in cells lacking the PTHrP NLS, there was a significant enrichment for genes that are upregulated in MCF7 cells overexpressing the oncoprotein and cell cycle promoter, cyclin D1 (Fig. 3B), indicating that the PTHrP NLS modulates the expression of cell cycle regulators to alter proliferation in MCF7 breast cancer cells. Furthermore, cells expressing PTHrP lacking both the NLS and C-terminal domain were positively enriched for genes involved in the p53 pathway (NES = 1.53, FDR = 0.053). We also examined enriched cancer Hallmark pathways, which revealed an increase in additional cell cycle-related pathways, including G2M Checkpoint and Mitotic Spindle genes (Fig. 3C&D).

Based on these RNA sequencing data which pointed to differences in genes encoding cell cycle regulatory proteins, and since p21 and p27 are known to be regulated downstream of PTHrP in other cell types [16–18], we investigated these cell cycle factors as a mechanism by which the PTHrP NLS and C-terminal domain oppositely influence breast tumor growth. Immunocytochemical staining revealed that while overexpression of full-length PTHrP (-36-139aa) did not alter p27 levels (Fig. 3E), p27 expression was significantly lower with deletion of the NLS alone compared to control cells. Furthermore, expression of p27 was significantly increased with deletion of both the NLS and C-terminal domain, exceeding levels in both MSCV controls and NLS-alone deleted cells (Fig. 3E). Immunofluorescent staining of the primary breast tumors similarly revealed no change in p27 with overexpression of the full-length PTHrP molecule, but p27 protein levels were significantly decreased with deletion of the NLS alone compared to controls, and oppositely increased with deletion of both the NLS and C-terminal domain (Fig. 3F). Interestingly, in vivo p27 protein levels still remained lower than controls with deletion of both domains (Fig. 3F). When we assessed p21 protein expression, we found inconsistent staining patterns between in vitro cultured cells and in vivo tumor sections; however, we did see a modest increase in p21 staining in tumors expressing full-length secreted PTHrP, suggesting p21 may be regulated downsteam of the intact PTHrP molecule in the context of the tumor microenvironment (Supplementary Fig. 3A&B). Together, these in vitro and in vivo findings suggest that p27 is oppositely regulated by the PTHrP NLS and C-terminal domain in breast cancer, with much lower levels in fast-growing tumors. The difference in p27 expression may therefore contribute to the differential proliferation and breast tumor growth effects observed in vivo.

### PTHrP regulates downstream LIFR signaling to alter p27 expression in vitro

**Fig. 4:**
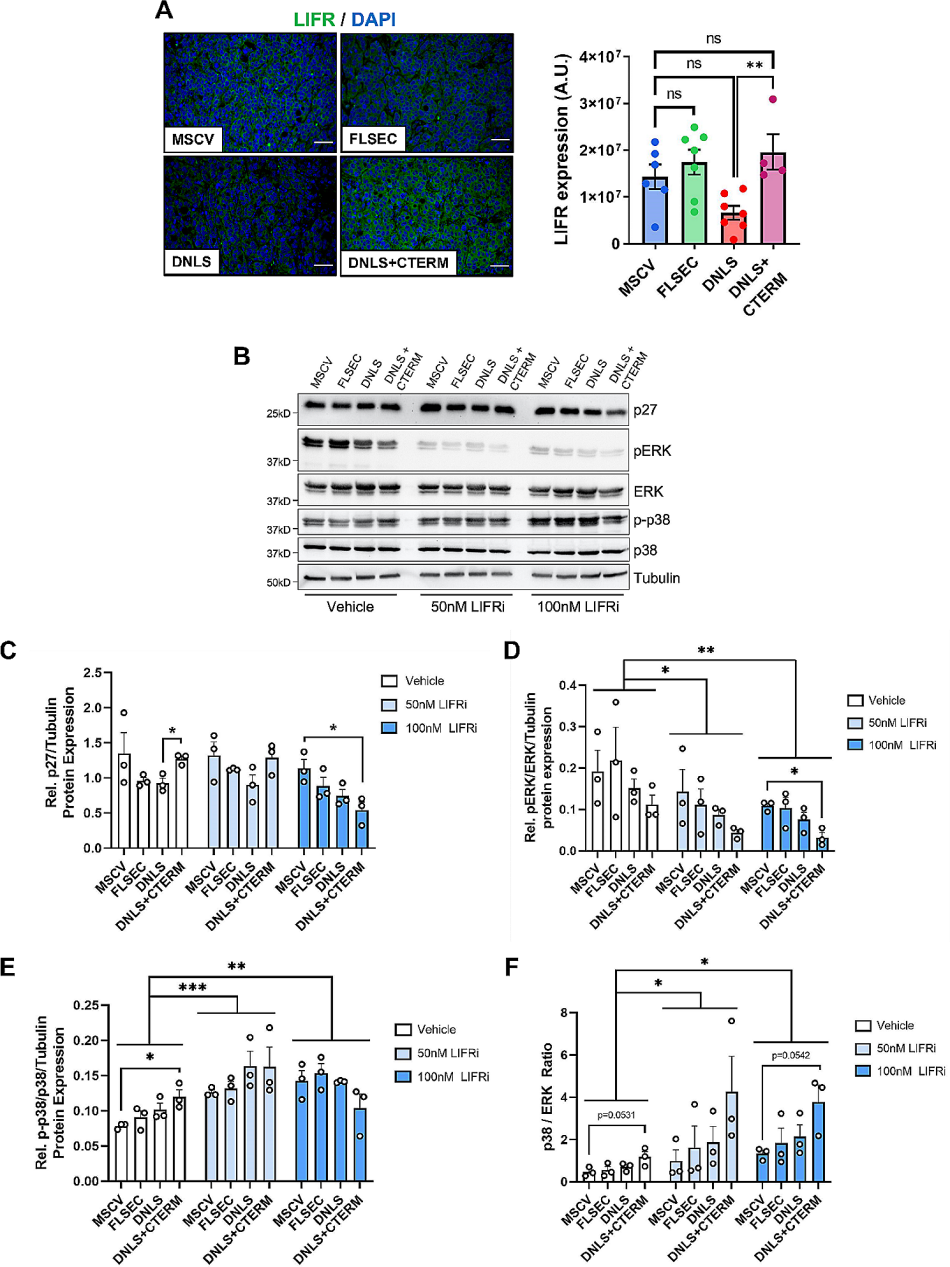
PTHrP differentially regulates p27 through LIFR in breast cancer cells. (**A**) Immunofluorescence staining and quantification of LIFR in primary tumors from mice inoculated with MSCV, FLSEC, DNLS, or DNLS + CTERM cells. All panels = 40X and scale bars = 50 *μ*m. (**B**) Western blot analysis of p27, pERK, ERK, p-p38, p38 and tubulin (loading control) protein levels in MSCV, FLSEC, DNLS, or DNLS + CTERM cells treated with vehicle (DMSO) or LIFR inhibitor (EC359, 50nM or 100nM) for 24 h. Densitometry for western blot analysis of (**C**) p27, (**D**) pERK/ERK and (**E**) p-p38/p38 described in (**B**). (**A**) **p < 0.01 vs. DNLS by unpaired t-test. (**C**) *p < 0.05 vs. DNLS by unpaired t-test or *p < 0.05 vs. MSCV by one-way ANOVA with multiple comparisons. (**D** & **E**) *p < 0.05 vs. MSCV by one-way ANOVA with multiple comparisons or *p < 0.05, **p < 0.01, ***p < 0.001 versus vehicle by two-way ANOVA. Graphs represent mean ± SEM

We previously demonstrated that PTHrP localizes to the proximal promoter region [40] and downregulates breast cancer cell expression of leukemia inhibitory factor receptor (LIFR) [32], which is a known breast tumor dormancy regulator in bone [32, 36], breast tumor suppressor, and lung metastasis suppressor [34, 35]. The downstream signaling mechanisms by which LIFR regulates breast tumor growth remain incompletely understood. While LIFR is a cell surface receptor, it can also be internalized to the cytoplasm once bound by the LIF ligand [41]. Although overexpression of full-length PTHrP (-36-139aa) has been shown to downregulate LIFR in vitro [32, 36, 38], we observed no difference in LIFR protein expression in vivo with overexpression of the full-length PTHrP molecule (Fig. 4A). Deletion of the PTHrP NLS alone modestly suppressed LIFR levels compared to MSCV controls while deletion of both the NLS and C-terminal domain significantly increased expression of LIFR compared to tumors lacking the NLS alone, which restored levels close to that of the control tumors (Fig. 4A). This pattern of increased LIFR expression with deletion of the PTHrP NLS and C-terminal domain (compared to NLS alone deletion) mirrored the previously observed trend in tumor p27 expression. Thus, we hypothesized that PTHrP may regulate tumor cell proliferation through p27 signaling downstream of LIFR, resulting in altered breast tumor cell proliferation.

To investigate this further, we treated the PTHrP mutant cells with a commercially available LIFR inhibitor (EC359) that blocks receptor/ligand interactions. Effective LIFR inhibition was confirmed by decreased phosphorylation of the downstream LIFR signaling factor, pERK (Fig. 4B & D). We did not observe changes in cell cycle phases with LIFR inhibitor treatment of the PTHrP mutant cells (Supplementary Fig. 4A). In the vehicle treated group, p27 remained significantly higher in cells expressing PTHrP lacking the NLS and C-terminal domain compared to those lacking the NLS alone (Fig. 4C). After 24 h of low dose LIFR inhibitor treatment (50nM), this difference was no longer significant (Fig. 4B & C). High dose treatment of LIFR inhibitor (100nM) for 24 h completely reversed the induction of p27 in cells lacking the PTHrP NLS and C-terminal domain such that p27 expression was significantly lower than even control MSCV cells (Fig. 4B & C). Together, these data suggest that PTHrP may induce p27 through a LIFR-dependent mechanism. Treatment of the PTHrP mutant cell lines with the LIFR inhibitor for 1 or 6 h did not elicit the same effect on p27 as the 24-h treatments, such that there was no change in the pattern of p27 protein levels compared with vehicle treated cells (Supplementary Fig. 4B-E). This lack of effect with shorter treatments suggests that p27 is likely an indirect downstream target of LIFR.

LIFR is a known dormancy regulator in breast tumor cells in the primary [32, 36, 38] and bone metastatic sites [32]. LIFR signaling activates multiple downstream signaling pathways in breast cancer, including ERK [42]. Since a high p38/ERK signaling ratio promotes tumor dormancy [43, 44], we also analyzed phosphorylated p38 levels in the PTHrP mutant cells, with and without LIFR inhibition. While phosphorylated p38 and the p38/ERK ratio were unchanged in the untreated cells expressing full-length or NLS alone-deleted PTHrP, both p38 and the p38/ERK ratio increased in cells expressing PTHrP lacking the NLS and C-terminal domain, compared to controls (Fig. 4D-F). This suggests that PTHrP lacking the NLS and C-terminal domain preferentially activates p38 signaling, which may induce a more quiescent phenotype. This is consistent with the significantly reduced primary tumor growth (Fig. 2A-C, DNLS + CTERM group). Interestingly, there was a significant increase in phosphorylated p38 and the p38/ERK ratio in the LIFR inhibitor treated cells compared to vehicle treated cells (Fig. 4F). This suggests that the LIFR inibitor may preferentially decrease ERK signaling, which in turn increases p38 activity.

### Loss of the PTHrP NLS enhances bone metastasis formation despite persistently elevated p27 expression

**Fig. 5:**
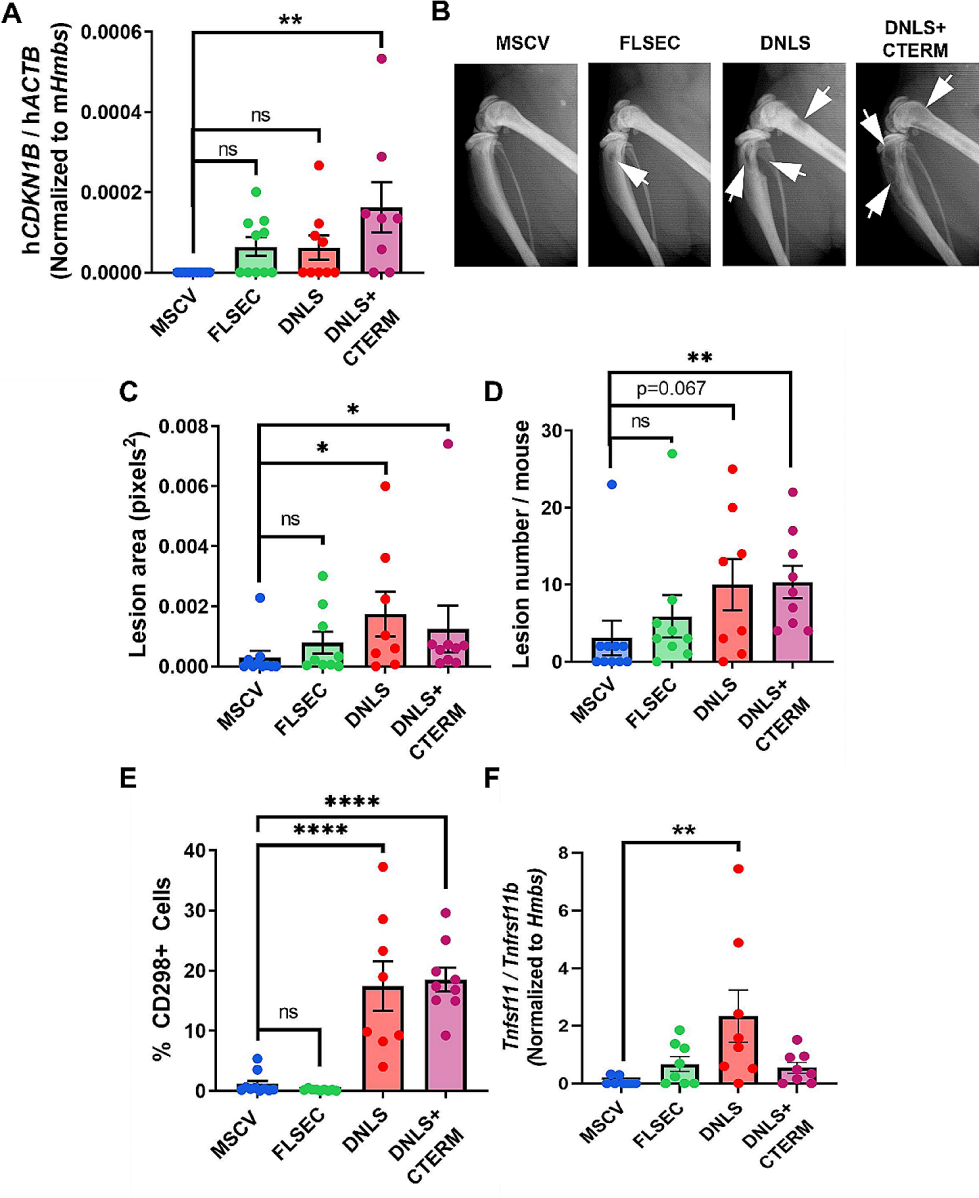
Truncated. PTHrP induces *CDKN1B* in the bone metastatic site, but enhances osteolysis and tumor burden. (**A**) qPCR analysis for *CDKN1B* (p27) normalized to *ACTB* as a marker of total tumor burden in the bone marrow of mice inoculated with MSCV, FLSEC, DNLS, or DNLS + CTERM cells via intracardiac injection. *n* = 8–10 mice/group. (**B-D**) Total osteolytic lesion area and lesion number (per mouse) based on radiographic analyses for mice described in A. White arrows indicate osteolytic lesions. (**E**) Flow cytometric quantitation of percent CD298 + tumor cells in the bone marrow of mice described in A. (**F**) qPCR analysis for RANKL/OPG (*Tnfsf11* / *Tnfrsf11b*) in whole homogenized femurs from mice described in (A). *n* = 8–10 mice/group. *p < 0.05, **p < 0.01, or ****p < 0.0001 vs. MSCV by one-way with multiple comparisons. Graphs represent mean ± SEM

Given the well-established role of PTHrP in promoting metastasis formation [8–11], we investigated how the NLS and C-terminal domain alter signaling and behavior of bone-disseminated tumor cells using a mouse model of bone colonization in which the PTHrP mutant tumor cells were inoculated through the left cardiac ventricle. We specifically examined whether elevated p27 expression is sustained in bone-disseminated breast tumor cells that express PTHrP lacking the NLS and C-terminal domain and if this alters proliferation, as in the primary tumor. Four weeks post-intracardiac inoculation, qPCR was performed on homogenized femora for human *CDKN1B* (gene name for p27), and normalized to *ACTB* (human tumor housekeeping gene) and *Hmbs* (mouse housekeeping gene) to quantify p27 specifically in bone-disseminated human tumor cells. *CDKN1B* was significantly higher in the homogenized femora from mice with bone-disseminated tumor cells that expressed PTHrP lacking the NLS and C-terminal domain only (Fig. 5A), confirming that even in the distant metastatic site, the truncated form of PTHrP induces more p27 in tumor cells than other PTHrP peptides. We observed the same trend in p27 expression in the primary tumor.

Surprisingly, although p27 levels were higher in the homogenized femora of mice inoculated with tumor cells that express PTHrP lacking the NLS and C-terminal domain, there was significantly elevated osteolytic bone destruction (Fig. 5B-D) and tumor burden (Fig. 5E) in the contralateral limb, as measured by flow cytometric analysis of CD298 + tumor cells, a validated marker for human tumor cells in the bone marrow [45]. The level of metastatic tumor growth and bone destruction was similar in mice inoculated with tumor cells expressing PTHrP either lacking the NLS alone or the NLS and C-terminal domain. This was in striking contrast to the primary tumor site where these cell lines expressing truncated forms of PTHrP elicited opposite effects on breast tumor growth (Fig. 2A-C). Thus, when the NLS and C-terminal domains are deleted, PTHrP induction of p27 persists in the bone metastatic site. However, in contrast to the primary tumor, induction of p27 downstream of PTHrP in disseminated tumor cells is not sufficient to prevent colonization of the bone and metastatic outgrowth, both of which are elevated by truncated PTHrP peptides lacking the NLS. To determine whether the increase in tumor burden was due to increased osteoclast-mediated bone resorption, we assessed the RANKL/OPG ratio in whole, homogenized femurs across all groups as a marker of osteoclasts. Surprisingly, we only observed a significant increase in RANKL/OPG when the PTHrP NLS domain was deleted, and not in the NLS + C-terminal deleted group. These data suggest that loss of the PTHrP NLS stimulates osteoclast-mediated bone resorption, but loss of the PTHrP NLS and C-terminus does not.

We also examined liver histological sections for metastatic tumor burden, but there were no lesions observed in any of the groups. Furthemore, in vitro we observed no difference in migratory potential of cells expressing full-length PTHrP or its truncated forms versus control cells (Supplementary Fig. 5). Together, these data suggest the PTHrP NLS and C-terminal domains may selectively enhance the ability of breast cancer cells to colonize, survive and proliferate specifically in the bone rather than broadly affecting their ability to migrate from the primary tumor and disseminate to other organs.

## Discussion

PTHrP is a critical driver of tumor-induced bone disease and an important regulator of breast tumorigenesis, cancer progression, and tumor dormancy [28, 32, 46, 47]. Here we investigated the intracellular actions of PTHrP through its NLS and C-terminal domain in breast cancer progression. An important finding is that deletion of the classical PTHrP NLS (67-94aa) does not preclude entry of PTHrP into the nucleus. This indicates that the truncated PTHrP peptides can translocate into the nucleus independent of this recognized NLS. Indeed, one study has reported that PTHrP (1-141) can be endocytosed and translocated into the nucleus via a non-PTH1R cell surface receptor [48], though the mechanism has not been fully elucidated. We are actively investigating alternative mechanisms by which PTHrP enters the nucleus when the classical NLS is deleted. These findings indicate that our study outcomes are likely due to differences in the binding partners or direct interactions of truncated PTHrP with other molecules, rather than the subcellular localization of the truncated peptides. We are also further investigating how the intracellular location alters binding partners of truncated PTHrP peptides to regulate downstream breast cancer cell signaling.

Our data demonstrate that the biological domains of PTHrP have distinct functions in breast cancer. These findings are consistent with studies from the skeletal field, which ascribe multiple biological functions to PTHrP domains, particularly through regions outside of the PTH1R-binding domain. Indeed, a knock-in mouse model (*Pthrp*D*/*D) lacking the midregion, NLS, and C terminal domain (67-137aa) revealed that the intracrine actions of PTHrP are crucial for normal skeletal development and the differentiation of osteogenic and hematopoietic precursors [49]. Most *Pthrp*D*/*D mice exhibit severe skeletal abnormalities, growth retardation, and die within 5 days. Injection with exogenous PTHrP fails to rescue the lethal phenotype providing further evidence that the effects of PTHrP on these physiological processes are primarily mediated by intracrine signaling. Another in vivo study demonstrated that knock-in mice expressing truncated PTHP (1-84aa) display abnormal skeletal growth and early lethality due to decreased cell proliferation, early senescence, and increased apoptosis in multiple tissues [16–18]. Together, these studies demonstrate the importance of the PTHrP NLS and C-terminal domain in regulating tissue development via intracrine signaling, and our data now identify distinct functions of these domains in the pathologic setting of breast cancer.

While a large body of evidence indicates that PTHrP has deleterious effects during late stages of breast cancer by promoting bone metastasis, tumor-induced osteolysis, and exit from dormancy, PTHrP’s role early in disease progression is highly controversial [27, 28, 32, 46, 47]. Prior preclinical studies reported directly conflicting evidence suggesting that PTHrP inhibits primary breast tumorigenesis in some models [27], while promoting tumor growth in others [28]. Our in vivo findings offer interesting insight into the complex role that PTHrP plays in breast tumor progression. Our data indicate that PTHrP lacking its classical NLS sequence dramatically accelerates breast tumor growth and proliferation in the primary tumor site, suggesting that this domain actually functions to suppress breast tumor growth. Surprisingly, this phenotype is completely reversed if breast cancer cells express PTHrP lacking both the NLS and C-terminal domain, suggesting that the C-terminal domain may possess oncogenic activity that opposes the influence of the NLS. Thus, we are actively pursuing studies to determine how expression or deletion of the C-terminus alone impacts breast cancer growth and bone colonization. Importantly, our data shed light on the conflicting preclinical studies suggesting that PTHrP can promote or inhibit breast tumorigenesis. These controversies may be in part due to the presence of different predominant truncated peptides of PTHrP containing the NLS or C-terminal domain. Unfortunately, these forms are not discernible by commercially available amino-terminal antibodies.

While studies have not identified the same engineered fragments as in our model presented here, it is feasible that fragments lacking the classical NLS (67-94aa) or the NLS and C-terminal domain (107-139aa) may naturally circulate in pre-clinical mouse tumor models and patients. In fact, the PTHrP sequence has numerous known and putative mono- and multi-basic cleavage sites [4, 50]. Importantly, PTHrP peptides containing the N-terminal domain (1-36aa), mid-regions (38-94aa), (38-95aa) and (38-101aa), as well as the C-terminal domain (107-139aa) have been detected in preclinical mouse models [21, 51] and from the plasma and urine of human patients with solid tumors [21, 51]. While very few studies have investigated a role for these and other PTHrP fragments in breast cancer, some limited studies have identified how their expression alters breast tumor cell behavior, breast tumor growth, and patient outcomes. The PTHrP mid-region fragment (38-94aa) containing a portion of the classical NLS is reported to inhibit in vitro proliferation of MDA-MB-231 human breast cancer cells [52] while another fragment from amino acids 87–106 reportedly stimulates proliferation in vitro [53]. In patients with breast cancer, loss of nuclear localized but not cytoplasmic PTHrP in the primary site has been associated with poor clinical outcomes [54]. Another study identified PTHrP (12–48) as a predictive biomarker of breast cancer bone metastasis such that levels of the peptide were significantly increased in the plasma of patients with clinical evidence of bone metastases versus patients without [55]. Together, these studies provide further evidence of domain-specific selectivity for how PTHrP and its truncated isoforms function in vitro versus in vivo.

While there were no changes in cell cycling observed in vitro, our in vivo studies demonstrate a modest increase in proliferation with deletion of the NLS alone, which persisted in the primary tumor but not bone. These differences in proliferation in vitro versus in vivo may also be attributed to PTHrP-induced signaling changes in the breast cancer cells that alter their interaction with surrounding stromal cells, including recruitment of immune cells into the tumor microenvironment, which vary substantially by tumor site. The present study sheds important light on the biological role for the classical NLS and C-terminal domain in regulating breast tumor growth in vivo.

Examination of cleaved PARP in the primary tumor demonstrated no alterations in apoptosis underlying the differences in tumor burden with expression of PTHrP lacking the NLS alone or both the NLS and C-terminal domain. We also examined levels of cleaved caspase-3 to more broadly assess apoptosis. One limitation in our model is that expression of caspase-3 is low at baseline in MCF7 cells, making it difficult to detect further reductions, particularly in cells expressing PTHrP lacking the DNLS. Importantly, the tumors assessed in our study were analyzed at endpoint, but it is possible that more dramatic changes in apoptosis occurred early in tumor progression. Indeed, the majority of tumors expressing PTHrP lacking the NLS and C-terminal domain were small in size and nearly undetectable at endpoint. The ability to measure apoptotic or proliferative markers from all tumors may have demonstrated a greater difference to further explain the alterations in tumor burden.

**Fig. 6:**
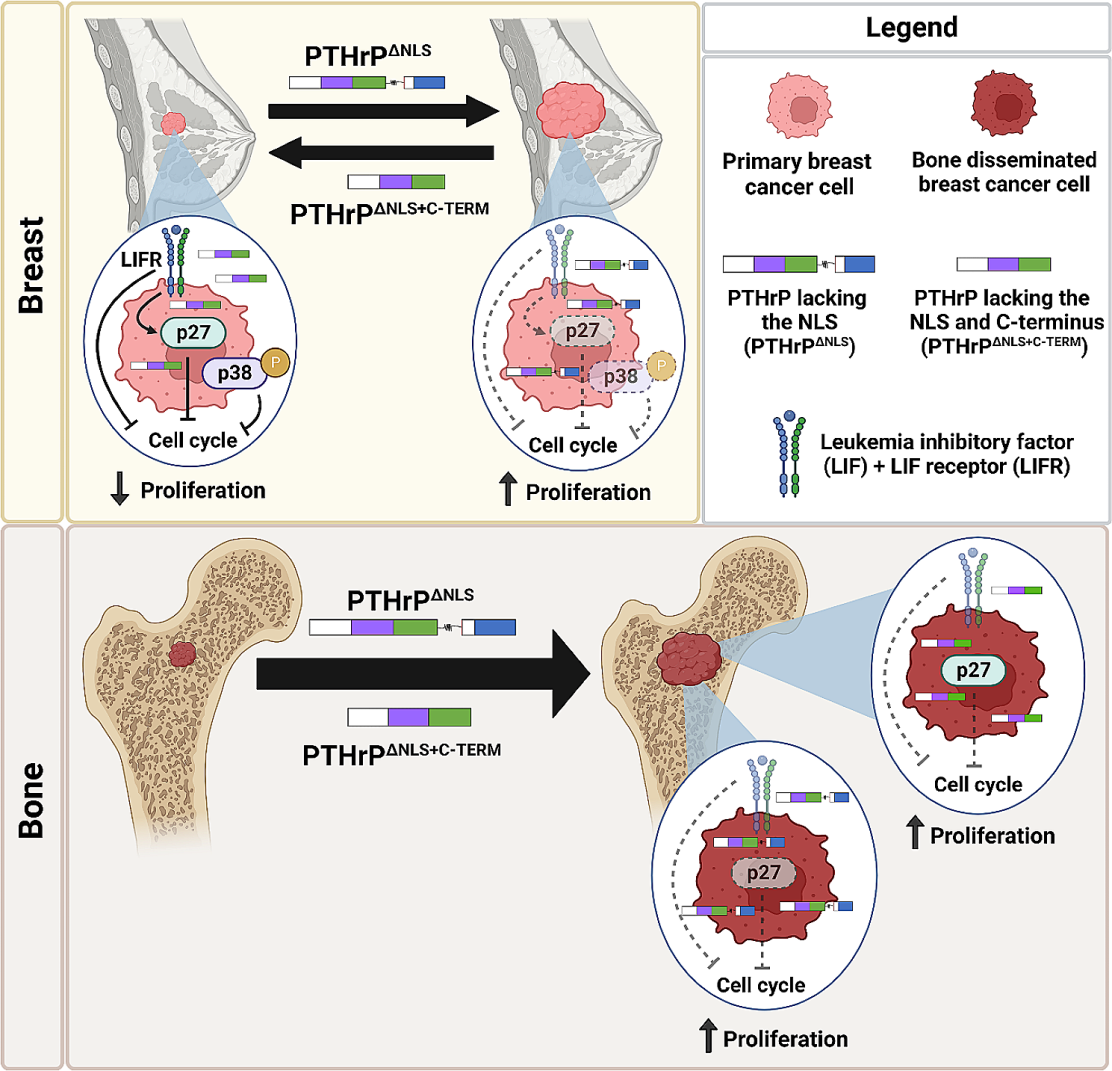
Model of PTHrP domain-specific actions in breast cancer progression and bone colonization. In the primary breast site (top left panel, left of arrows), PTHrP lacking the NLS and C-terminal domain decreases tumor cell proliferation through p27 induction driven by the tumor suppressor leukemia inhibitory factor receptor (LIFR). PTHrP lacking the NLS and C-terminal domain also preferentially induces p38 phosphorylation and signaling to inhibit cell cycling downstream of LIFR activation. In the breast, truncated PTHrP lacking the NLS alone (top left panel, right of arrows) downregulates LIFR expression (denoted by transparent coloring) and prevents induction of p27 expression and activation of p38 signaling (denoted by dashed arrows, dotted outlines and transparent coloring) to drive cell proliferation and tumor growth. In bone disseminated tumor cells (bottom panel), LIFR expression is downregulated and the induction of p27 by PTHrP lacking the NLS and C-terminal domain persists, but is not sufficient to repress metastatic outgrowth (denoted by dashed inhibitor line), in contrast to the primary tumor. In the bone, tumor cells expressing PTHrP peptides lacking the NLS or NLS and C-terminal domain readily proliferate into metastatic tumors. Image created with Biorender.com

Cyclin dependent kinase inhibitor proteins are regulated downstream of the PTHrP NLS and C-terminal domain in non-breast cancer cell lineages [16–18]. Our studies demonstrate that p27 is oppositely regulated by the PTHrP NLS and C-terminal domain in breast cancer and may be an important downstream signaling factor mediating how these domains differentially alter breast tumor growth (Fig. 6). Specifically, the PTHrP C-terminal domain appears to function as an oncogenic molecular switch able to induce proliferation and promote primary breast tumor formation through a partially LIFR-dependent mechanism that suppresses p27 expression. It should be noted that there are significant differences in tumor burden and p27 between control tumors and tumors expressing PTHrP that lack the NLS, but a non-significant decrease in LIFR (~ 50% reduction). Thus, the data are consistent across our in vivo study, but do not always result in statistically significant changes. This suggests that LIFR is not the only driver of p27 in our model. Future studies utilizing breast cancer cells expressing PTHrP with deletion of the C-terminal domain only will be needed to confirm this. Interestingly, although *CDKN1B* (gene name for p27) remained elevated by the bone-disseminated tumor cells expressing PTHrP lacking the NLS and C-terminal domain, the cells readily colonized the bone marrow. We thought this may be due to an increase in osteoclast-mediated bone resorption, which we assessed by measuring RANKL/OPG levels in whole, homogenized femora. We were surprised that RANKL/OPG was only elevated when the PTHrP NLS was deleted, and not when the NLS and C-termainal domain were deleted, since both groups had similar levels of bone destruction and bone metastatic tumor burden. This finding suggests that the mechanism of tumor outgrowth caused by the PTHrP fragments is likely distinct, and that the osteoclast-mediated osteolysis must have occurred early in disease progression in the tumors lacking the PTHrP NLS and C-terminus, since measurements were assessed at endpoint. Follow-up studies to identify the distinct mechanisms of tumor outgrowth in bone that are caused by each PTHrP fragment are underway.

In our studies, pharmacologic LIFR inhibition revealed an unexpected trend whereby breast cancer cells treated with the inhibitor had significantly elevated phosphorylated p38 and a p38/ERK signaling ratio compared to vehicle treated cells, regardless of PTHrP mutant expression. This effect was further elevated when the PTHrP NLS and C-terminal domain were deleted. LIFR is known to activate STAT3, ERK, and AKT signaling, among numerous other signaling pathways in breast cancer [32, 42, 56]. It has been postulated that LIFR signaling promotes tumor dormancy specifically through STAT3 activation [32]; however, the oncogenic ERK and AKT pathways can still be activated by LIFR-binding cytokines [42]. Our data here suggest that the EC359 LIFR inhibitor may preferentially decrease LIFR-mediated ERK signaling, shifting the balance towards p38 activity and suppression of cell proliferation in vitro. Since LIFR activates multiple singaling pathways in breast cancer cells [42], we also sought to analyze alterations in STAT3 and AKT signaling in the presence and absence of LIFR inhibition via western blot analysis; however, activation of these pathways was too low at baseline to quantify discernable changes in pSTAT3 and pAKT.

Recently, small molecule inhibitors and neutralizing antibodies targeting LIFR have been investigated as a strategy to inhibit breast tumor growth and metastasis in preclinical studies [57, 58]. Although anti-LIFR agents do show evidence of effectively limiting primary breast tumor growth, caution should still be exercised in their use as a breast cancer therapy since inhibiting LIFR signaling could inadvertently increase metastatic outgrowth in bone where the LIFR:STAT3 pathway suppresses proliferation of disseminated breast tumor cells [32, 59–61]. It will therefore be important to define the downstream pathways that are disrupted by individual LIFR antagonists. Furthermore, it is still unclear how the PTHrP NLS and C-terminal domains may differentially regulate other downstream LIFR signaling pathways.

## Concluding remarks

In summary, these data reveal important insights into how the PTHrP NLS and C-terminal domain divergently control breast cancer progression through p27 signaling in the primary tumor and bone metastatic site. As a potent regulator of breast tumor growth and distant metastatic progression, PTHrP has the potential to be leveraged as a therapeutic target for the treatment of breast cancer at multiple stages of disease progression and possibly for the prevention of bone metastasis formation. However, it is critical that this work be approached with attention to the PTHrP peptides present and their ability to differentially activate downstream signaling pathways.

## Materials and methods

### Cell culture and reagents

#### Cells

PTHrP mutant cell lines were established in the laboratory of one of us (TJM) at St. Vincent’s Institute of Medical Research, as previously described [61]. Briefly, the following constructs were synthesized by Integrated DNA Technologies (IDT) (Coralville, IA, USA): *Pthlh*(-36-139), *Pthlh* (1-139), *Pthlh*(-36-67), *Pthlh*(-36-139). Xho1/ EcoR1 enzyme digestion and ligation was performed to clone the constructs into the murine stem cell virus (MSCV)-zeo plasmid. Each plasmid except for the MSCV control was tagged with a human influenza hemagglutinin (HA) epitope at the C-terminal end. DNA sequencing was performed by the Australian Genome Research Facility. Phoenix cells were then transfected with the mutant plasmids and used to infect MCF7 cells which were placed under antibiotic selection with Zeocin to establish stable lines. The resulting PTHrP mutant cells were cultured in DMEM supplemented with 10% fetal bovine serum (FBS) and 1% penicillin/streptomycin (P/S). All cell lines were regularly tested for mycoplasma contamination.

#### Proliferation assays

Cells were plated at 1 × 10^6^ cells per 10cm^2^ plate and allowed to adhere for 4–6 h. Adherent cells were then trypsinized and mixed with 0.4% trypan blue solution. Viable cells were determined based on dye exclusion and counted using a TC20 Automated Cell Counter (Bio-Rad). Proliferation of PTHrP mutant cells was monitored daily for four days by trypsinizing and counting viable cells.

#### LIFR inhibitor treatment

Cells were plated at 1 × 10^6^ cells/ 10cm^2^ plate and allowed to adhere overnight. The following day, cells were treated with EC359, a leukemia inhibitory factor receptor (LIFR) inhibitor (50nM or 100nM; MedChemExpress; Catalog No. HY-1,201,420) or vehicle (0.1% dimethyl sulfoxide, DMSO) for 1, 6, or 24 h in full-serum media.

### RNA extraction and real-time qPCR

RNA was extracted from cells using TRIzol (ThermoFisher) and prepared for real-time qPCR analysis as previously described [32]. Human primers for b2M [32] and *CDKN1B* (p27) were previously published. The following primers were designed using PrimerBlast (NCBI) against the human genome and validated by dissociation: ACTB (F- CATGTACGTTGCTATCCAGGC), R- CTCCTTAATGTCACGCACGAT). Mouse primers for HMBS were previously published [32]. The following primers were designed using PrimerBlast (NCBI) against the mouse genome (Mus musculus) and validated by dissociation: PTHrP mid-region (F- CATCAGCTACTGCATGACAAGG, R- GGTGGTTTTTGGTGTTGGGTG), PTHrP NLS (F- AACAGCCACTCAAGACACCC, R- GACCGAGTCCTTCGCTTCTT), PTHrP C-terminal region (F- AAAAGAAGCGAAGGACTCGG, R- GCGTCCTTAAGCTGGGCT).

### Western blotting

Cultured cells were rinsed twice with cold 1X PBS and harvested in RIPA lysis buffer (Sigma) containing protease and phosphatase inhibitors (Roche). Protein lysate (20μg) was loaded onto an SDS-PAGE gel under reducing conditions and transferred to nitrocellulose membranes. Membranes were probed with antibodies against HA-Tag (Cell Signaling, C29F4, Catalog No. 37T4S, 1:1000), LIFR (Santa Cruz, C-19, Catalog No. sc-659, 1:1000), p21Waf1/Cip1(Cell Signaling, Catalog No. 2947 S, 1:1000), p27 Kip1 (Cell Signaling, Catalog No. 3686 S, 1:1000), phospho-p38 MAPK (Thr180/Tyr182) (Cell Signaling, Catalog No. 4511, 1:1000), p38 MAPK (Cell Signaling, Catalog No. 8690, 1:1000), phospho-ERK1/2 Thr202/Tyr204 (Cell Signaling, Catalog No. 9101, 1:1000), ERK1/2 (Cell Signaling, catalog number 9102, 1:1000), Calnexin (AbCam, Catalog No. ab22595-100UG, 1:900), GAPDH (Cell Signaling 14C10, Catalog No. 2118 S, 1:5000), HDAC2 (Cell Signaling, D6S5P, 1:1000), α-tubulin (Antibody & Protein Resource at Vanderbilt University, Catalog No. VAPRTUB, 1:5000), or Vinculin (Millipore, Catalog No. AB6039, 1:1000).

### Nuclear and cytoplasmic extraction

Nuclear and cytoplasmic extracts were obtained from cultured PTHrP mutant cells using the NE-PER Nuclear and Cytoplasmic Extraction Reagents Kit (Thermo Scientific, Catalog No. 78,835) according to the manufacturer’s instructions. Briefly, 5 × 10^6^ cells were plated in full serum DMEM and allowed to adhere overnight. The following day, adherent cells were trypsinized and centrifuged at 500 x *g* for 5 min, and the pellet was suspended in PBS. Cells were then transferred to a new microcentrifuge tube and centrifuged at 500 x *g* for 3 min. Supernatant was discarded and 500 µl of ice-cold CER I with 5 µl of protease inhibitor was added to the cell pellet and vortexed. The cell suspension was incubated on ice for 10 min. Ice-cold CER II (27.5 µl) was then added to the tube, vortexed, and incubated on ice for 1 min. Next, the sample was vortexed and centrifuged at 16,000 x *g* for 5 min. The supernatant (cytoplasmic extract) was immediately transferred to a clean pre-chilled tube and stored at -80^o^C. The cell pellet was suspended in 250 µl of ice-cold NER, vortexed for 15 s, and placed on ice. Vortexing was repeated every 10 min for a total of 40 min. The tube was then centrifuged at 16,000 x *g* for 10 min. Finally, the supernatant (nuclear extract) was transferred to a clean pre-chilled tube and stored at -80^o^C.

### Immunocytochemistry

For analysis of HA-tagged PTHrP peptides, cells were seeded onto a 4-well culture slide at 6 × 10^5^ cells/ well and allowed to adhere overnight. The following day cells were washed twice with 1x PBS and fixed with 10% formalin for 15 min. Cells were then washed three times with 1X PBS for 5 minutes each, permeabilized in 0.25% Triton-X in 1X PBS for 10 min and washed twice with 1X PBS for 5 minutes each. Next cells were blocked in a 3% mix of donkey horse serum (DHS)/ bovine serum albumin (BSA) for 1 h at room temperature, washed twice with 1X PBS for 5 minutes each and finally incubated with HA-Tag antibody (Cell Signaling, C29F4, Catalog No. 37T4S, 1:500) diluted in DHS/ BSA mix for 1 h at room temperature. Afterwards, cells were washed three times with 1X PBS for 5 minutes each and incubated in goat anti-rabbit IgG (H + L) Alexa Fluor 488 secondary antibody (Thermo Fisher, Catalog No A-11,034, 1:1000) diluted in DHS/ BSA mix in the dark for 1 h at room temperature. Cells were then washed three times with 1X PBS for 5 minutes each. Lastly, the chamber was removed from each slide before mounting coverslips with VECTASHIELD HardSet Antifade Mounting Medium with DAPI (Vector Laboratories). Fixed cells were imaged on a laser scanning confocal microscope Nikon A1r based on a TiE motorized Inverted Microscope using a (I) 60X lens, NA 1.4, run by NIS Elements C software with sections imaged in 0.23 μm slices or (II) 100X lens, NA 1.49, run by NIS Elements C software with sections imaged in 0.23 μm slices.

For analysis of p21 and p27, 8 × 10^5^ cells were seeded onto glass coverslips coated with 5 µg/ml human fibronectin (Millipore) 1–2 h prior. The following day, cells were washed with 1X PBS, fixed with 10% formalin for 15 min, washed three times with 1X PBS for five minutes each and permeabilized with 0.25% Triton-X for 10 min. Afterwards, cells were washed twice with 1X PBS for 5 minutes each and blocked with DHS/ BSA mix for 1 h at room temperature. Cells were then washed twice with 1X PBS for 5 minutes each and incubated in p21Waf1/Cip1(Cell Signaling, Catalog No. 2947 S, 1:1000) or p27 Kip1 (Cell Signaling, Catalog No. 3686 S, 1:1000) diluted in DHS/BSA mix for 1.5 h at room temperature. Afterwards cells were washed three times with 1X PBS for 5 minutes each and incubated in goat anti-rabbit IgG (H + L) Alexa Fluor 488 secondary antibody (Thermo Fisher, Catalog No A-11,034, 1:1000) diluted in DHS/ BSA mix in the dark at room temperature. Finally, cells were washed three times with 1X PBS for 5 minutes each before mounting on glass slides with VECTASHIELD HardSet Antifade Mounting Medium with DAPI (Vector Laboratories). Images were collected on an Olympus BX41 Microscope equipped with an Olympus DP71 camera using the 40X plain objective. For p21 quantitation in Image J, total nuclei and positive staining cells were counted manually to calculate the percent of positive staining cells. For p27, the fluorescence intensity was quantified using ImageJ with manual cell contouring and measurement of the Raw Integrated Density which was averaged across all cells from 3 separate images.

### Enzyme-linked immunosorbent assay

To prepare conditioned media, PTHrP mutant cells (1 × 10^5^) were plated in full-serum media in a 24-well plate and allowed to adhere for 24 h. Afterwards, the full-serum media was changed to 600 µl of reduced serum media (DMEM + 2% FBS + 1% P/S) and cells were incubated for 24 h. Conditioned cell media was then harvested and centrifuged at 1500 rpm for 10 min at 4 °C. The supernatant was treated with protease inhibitor (Sigma, P8340, 1:100) before further analysis. Undiluted conditioned media was added to 96-well ELISA plates to measure secreted PTHrP levels according to the manufacturer’s protocol (Creative Diagnostics, Catalog No. DEIA2034). For the final analysis, calculated PTHrP concentrations measured by the ELISA were normalized to the total protein concentration (mg/ml) in each sample measured by BCA assay (Thermo Fisher).

### Cell cycle analysis

Cell cycle analysis was performed by seeding 150,000 cells per well into 6-well plates for each cell line. After 24 h, cells were treated with 50nM EC359, 100nM EC359, or DMSO vehicle for 48 h. After 48 h, 150,000 cells were removed from each treatment group and live stained with Hoescht 33342 (AbCam) at a concentration of 10 µg/mL for 1 h at 37 °C. Stained cells were analyzed on a 4 Laser Fortessa by the Vanderbilt Flow Cytometry Resource Core. Flow cytometer data were analyzed using FlowJo software to gate for G0/1, S, and G2 phases. Each bar represents data from 3 independent experiments.

### Migration assay

Scratch assays were performed by seeding 400,000 cells of each mutant cell line (MSCV, FLSEC, DNLS, and DNLS + CTERM) into one well of a 6-well plate. After 24 h, three scratches were made in each well with a pipette tip. Images were taken at 100x on an inverted microscope at 0 h (immediately after scratch), 24 h, and 48 h. Percent closure was determined via analysis with ImageJ. Each replicate is expressed as an average of three scratches per well. Each data point represents three independent experiments.

### Animal studies and imaging

#### Animals

Experiments were performed under the regulations of the Animal Welfare Act and the Guide for the Care and Use of Laboratory Animals and approved by the Vanderbilt University Institutional Animal Care and Use Committee (IACUC). For the mammary fat pad study, 17β-estradiol pellets (0.36 mg/pellet; Innovative Research of America, Catalog No. SE-121) were subcutaneously implanted into female athymic nude mice 24 h prior to tumor inoculation [61]. The following day, 5 × 10^5^ tumor cells from each pooled cell line in 20 µl PBS + 50% matrigel (Fisher Scientific) were inoculated into the fourth mammary fat pad (*n* = 10 mice injected per group). Tumor volume was assessed by caliper measurement. Several mice had to be sacrificed early due to estrogen-induced toxicities resulting in MSCV = 8 mice, FLSEC = 7 mice, DNLS = 10 mice, DNLS + CTERM = 9 mice in the final analysis. For the intracardiac inoculation study, 6-week-old female athymic nude mice (Jackson, Catalog No. 7850) were injected with 1 × 10^5^ tumor cells from each pooled cell line as previously described [63] (*n* = 8–10 mice injected per group). The mice were subcutaneously implanted with a slow-release 17β-estradiol pellet (0.36 mg/pellet; Innovative Research of America, Catalog No. SE-121) 24 h prior to tumor cell injection [63].

#### Radiography

Radiographic (x-ray) images were obtained as previously described [64]. Briefly, a Faxitron LX-60 (34 kV for 8 s) was used to acquire x-ray images and images were quantified for osteolytic lesion number and area using ImageJ software.

#### Histology

Upon sacrifice of the mice, dissected tumors were fixed in 10% formalin for 48 h and stored in 70% ethanol until being paraffin-embedded for further analyses. Tissue sections were deparaffinized by heating the slides to 50 °C and placed in xylene for 5 min and then 3 min. Next, slides were soaked in 100%, 95%, and then 75% ethanol for 3 min each. Slides were slowly changed to deionized water and rinsed twice in water. The slides were immersed in 10 mM TRIS (pH 9.0) and 1 mM EDTA heated to 150 °C for 20 min. After cooling at room temperature for 20 min, slides were rinsed twice with water and then three times with 1X PBS followed by blocking with 10% BSA in PBS for 2 h. Sections were stained with Ki67 (Thermo Fisher; Catalog No. RM9106S0, 1:500), cleaved PARP (Asp214) (Cell Signaling Technology, Catalog No. 5625T, 1:500), HA-Tag (Cell Signaling, C29F4, Catalog No. 37T4S, 1:1000), p21Waf1/Cip1(Cell Signaling, Catalog No. 2947 S, 1:1000), or p27 Kip1 (Cell Signaling, Catalog No. 3686 S, 1:1000) in 3% BSA in PBS overnight at 4 °C. The following day, sections were washed three times with 1X PBS and incubated in goat anti-rabbit IgG (H + L) Alexa Fluor 488 secondary antibody (Thermo Fisher, Catalog No A-11,034, 1:1000) in 3% BSA/PBS in the dark at room temperature for 1 h. Finally, sections were washed three times with 1X PBS and coverslips were mounted using VECTASHIELD HardSet Antifade Mounting Medium with DAPI (Vector Laboratories). For LIFR staining, after blocking in 10% BSA for 2 h, slides were incubated in FITC-LIFR (Santa Cruz, Catalog No. sc-515,337, 1:50) in 3% BSA/PBS overnight at 4 °C. The following day, sections were washed three times with 1X PBS and coverslips mounted using VECTASHIELD HardSet Antifade Mounting Medium with DAPI (Vector Laboratories).

All images except for Ki67 were collected on an Olympus BX41 Microscope equipped with an Olympus DP71 camera using the 40X plain objectives. For LIFR quantitation, 40X images were used and an area measuring 1900 × 1180 pixels was selected to measure the Raw Integrated Density. The Raw Integrated Density from 3 representative images was averaged for each mouse and these values are reported in the figure. For p21, p27, and cleaved PARP, the quantitation was performed using ImageJ analysis of the 40X images. Positive staining nuclei and total cell counts were determined using color thresholding in ImageJ and the number of positive staining nuclei was divided by the total number of nuclei present to calculate the percent positivity. For Ki67 quantification, fixed samples were imaged on a laser scanning confocal microscope Nikon A1r based on a TiE motorized Inverted Microscope using a 60X lens, NA 1.4, run by NIS Elements C software. Sections were imaged in 0.4 μm slices. Positive staining nuclei and cell counts were determined using color thresholding in ImageJ and the number of positive staining nuclei was divided by the total number of nuclei present to calculate percent Ki67 positivity.

#### Flow Cytometry

One hindlimb (inclusive of bone marrow and tumor cells) was crushed with a mortar and pestle to obtain the bone marrow. PBS (1mL) was added to the crushed bone marrow and spun down and washed with PBS to remove bone debris. Bone marrow (5 × 10^5^ cells) was stained in 100µL of PBS with LIVE/DEAD™ Fixable Green Dead Cell Stain Kit @488nm (Thermo Fisher Scientific, Catalog Number L34970, 1:1000) for 15 min on ice at 4 °C in the dark. Cells were washed with PBS and resuspended with 100µL of 1% BSA in PBS with CD298 antibody (BioLegend, Cat #341,704) for 30 min on ice at 4 °C in the dark.

#### Flow Cytometry Analysis

Flow cytometry experiments were performed in the VUMC Flow Cytometry Shared Resource using the 5-laser BD LSRII and 4-laser BD Fortessa LSRII. Data was analyzed using FlowJo software (FlowJo, LLC) where bone marrow samples were gated based on forward scatter and side scatter geometry, and PE-CD298 (+) cells were gated using live cells (LIVE/DEAD-Green negative) as previously validated in tumor-bearing bone marrow samples [45]. MCF7 breast cancer cells were used as a positive control for CD298 stain.

### Statistics and reproducibility

For all experiments, *n* per group is as indicated by the figure legend and the scatter dot plots indicate the mean of each group and error bars indicate the standard error of the mean. All graphs and statistical analyses were generated using Prism software (Graphpad). Statistical significance for all in vitro and in vivo assays was analyzed using an unpaired t-test, one-way ANOVA with Sidak’s multiple comparisons test or two-way ANOVA with multiple comparisons, as indicated in the figure legends. For each analysis *p* < 0.05 was considered statistically significant, and *p < 0.05, **p < 0.01, ***p < 0.001, ****p < 0.0001.

### Electronic supplementary material

Below is the link to the electronic supplementary material.

Supplementary Material 1

Supplementary Material 2

Supplementary Material 3

## Data Availability

Data that support the findings of this study are available from the corresponding author upon reasonable request.
