# Supplementary material for: PTHrP intracrine actions divergently influence breast cancer growth through p27 and LIFR
Source: Breast Cancer Res. 2024 Feb 26;26:34. doi: 10.1186/s13058-024-01791-z (PMC10897994; doi:10.1186/s13058-024-01791-z)
Supplement: Supplementary file 2 — Supplementary Material 2 [file 13058_2024_1791_MOESM2_ESM.pptx]

## Slide 1
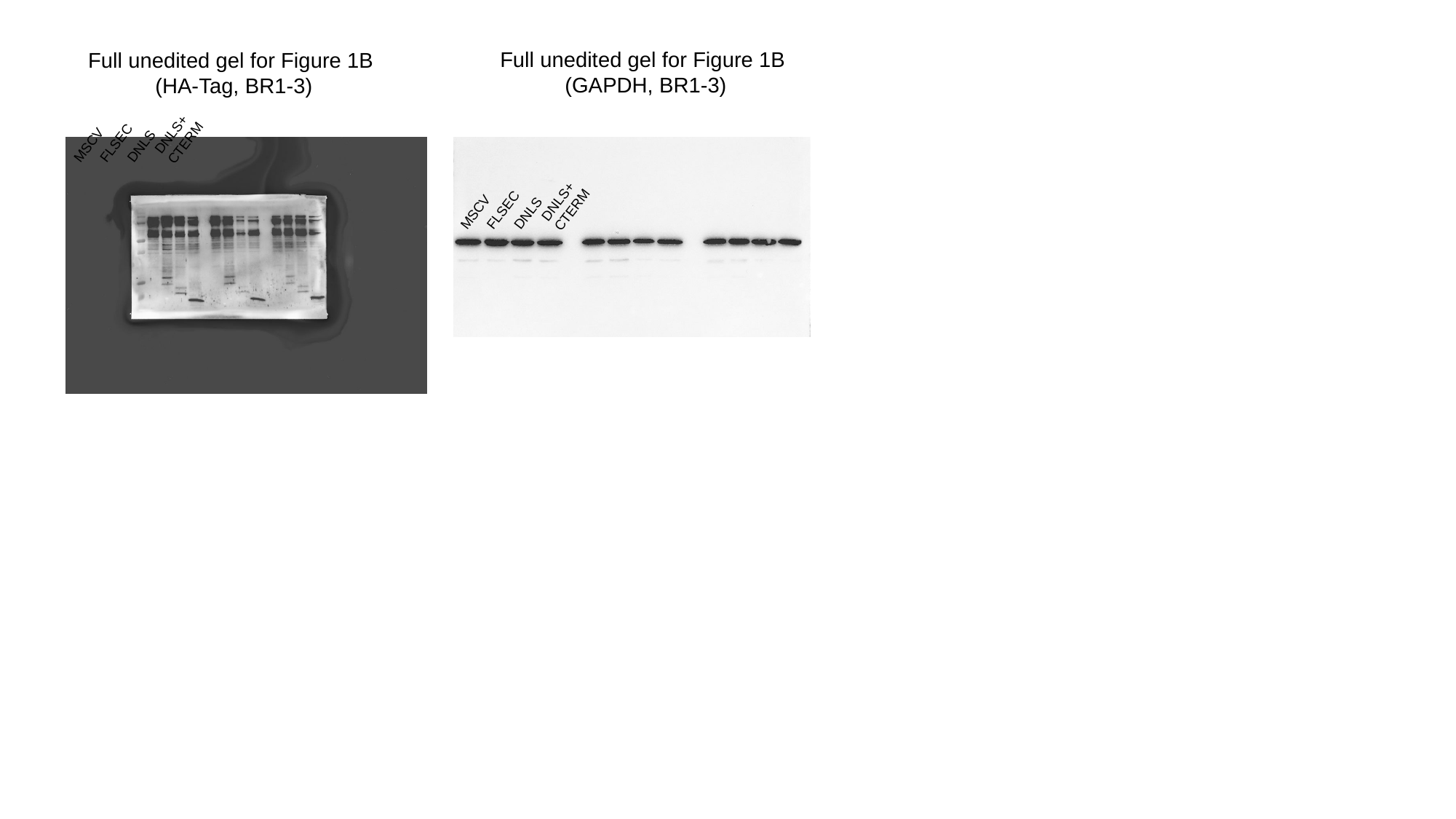

Full unedited gel for Figure 1B
(GAPDH, BR1-3)
Full unedited gel for Figure 1B
(HA-Tag, BR1-3)
DNLS+
CTERM
FLSEC
MSCV
DNLS
DNLS+
CTERM
FLSEC
MSCV
DNLS

## Slide 2
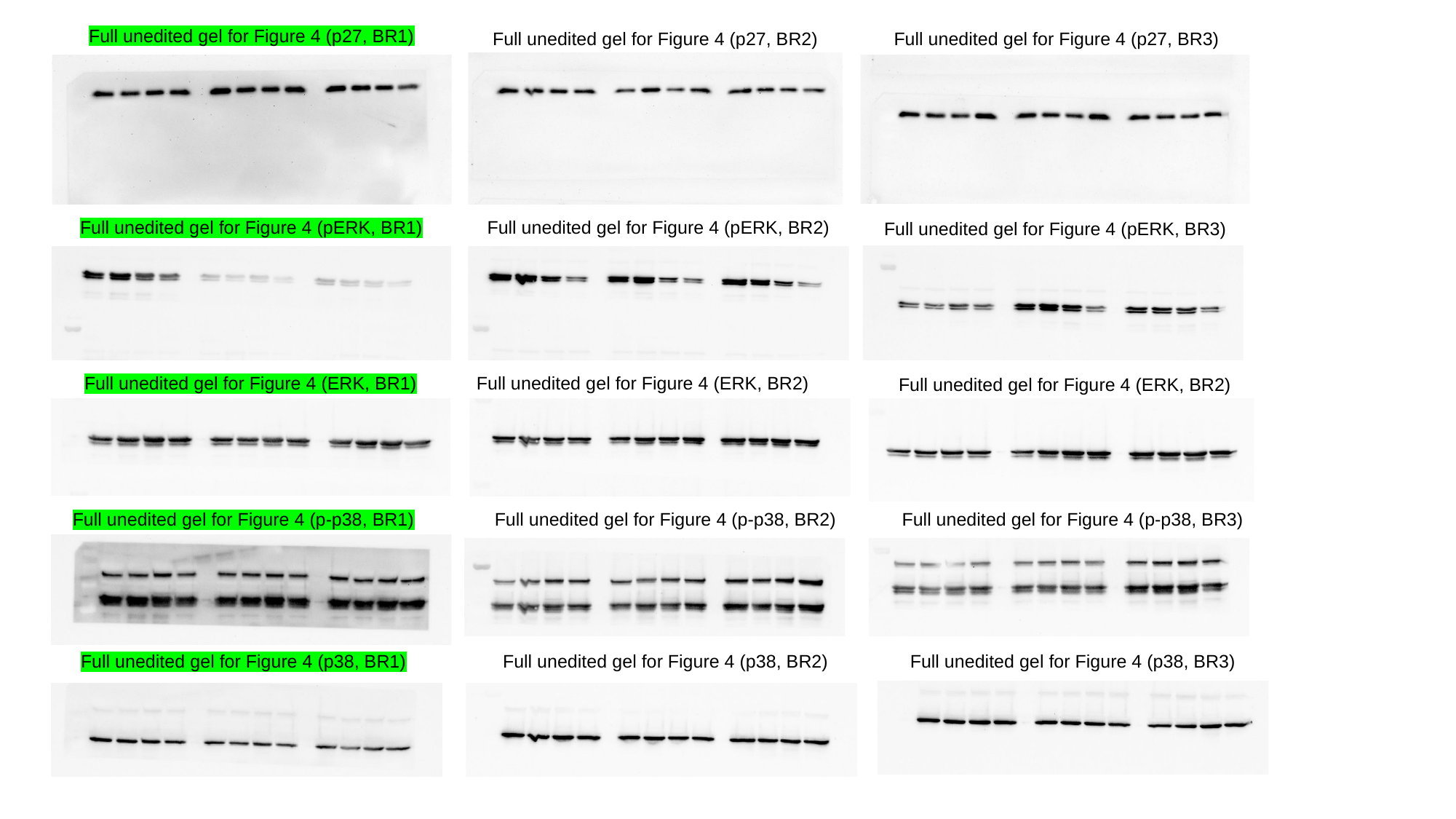

Full unedited gel for Figure 4 (p27, BR1)
Full unedited gel for Figure 4 (p27, BR2)
Full unedited gel for Figure 4 (p27, BR3)
Full unedited gel for Figure 4 (pERK, BR1)
Full unedited gel for Figure 4 (pERK, BR2)
Full unedited gel for Figure 4 (pERK, BR3)
Full unedited gel for Figure 4 (ERK, BR2)
Full unedited gel for Figure 4 (ERK, BR1)
Full unedited gel for Figure 4 (ERK, BR2)
Full unedited gel for Figure 4 (p-p38, BR1)
Full unedited gel for Figure 4 (p-p38, BR2)
Full unedited gel for Figure 4 (p-p38, BR3)
Full unedited gel for Figure 4 (p38, BR1)
Full unedited gel for Figure 4 (p38, BR2)
Full unedited gel for Figure 4 (p38, BR3)

## Slide 3
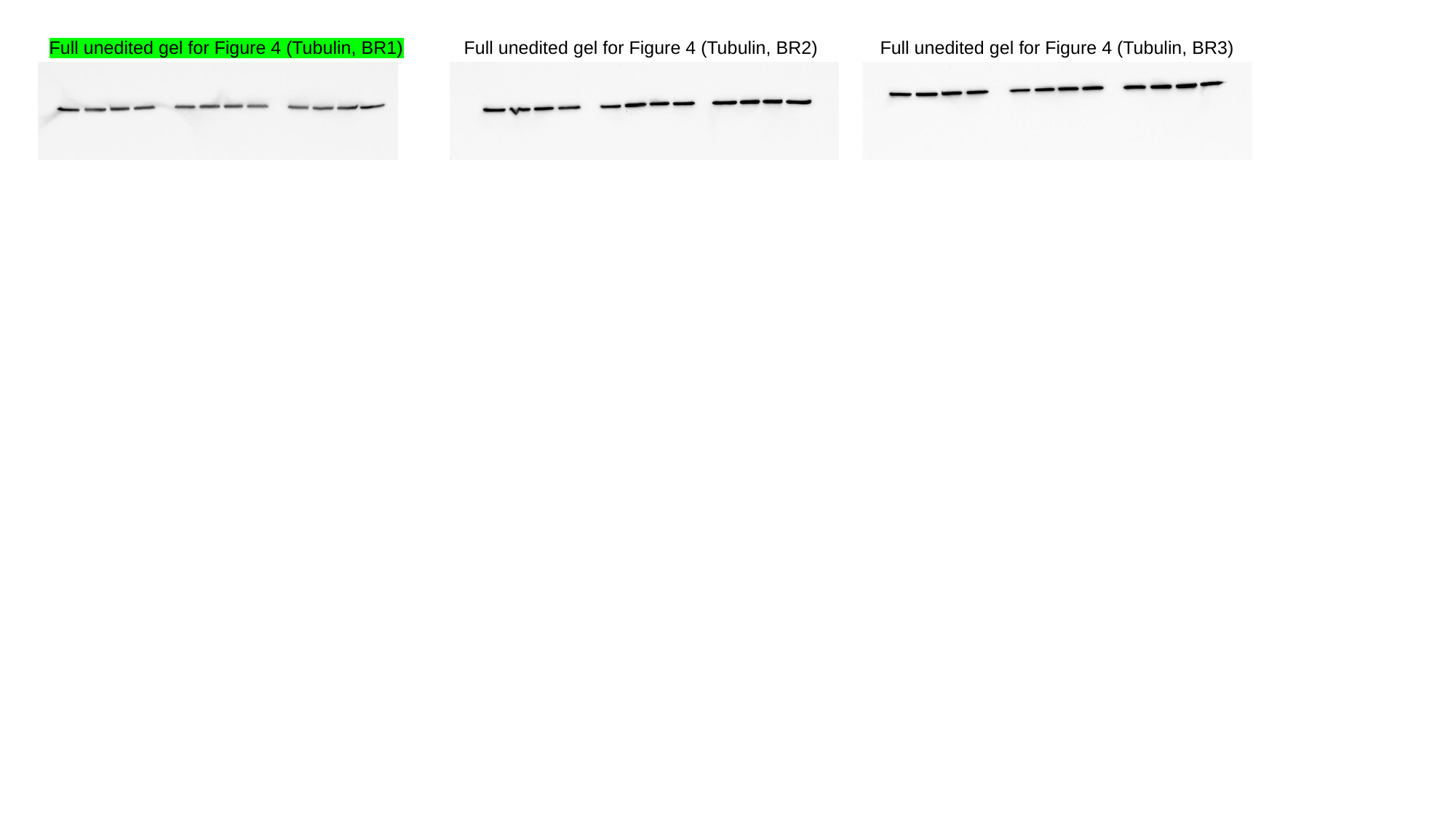

Full unedited gel for Figure 4 (Tubulin, BR3)
Full unedited gel for Figure 4 (Tubulin, BR2)
Full unedited gel for Figure 4 (Tubulin, BR1)

## Slide 4
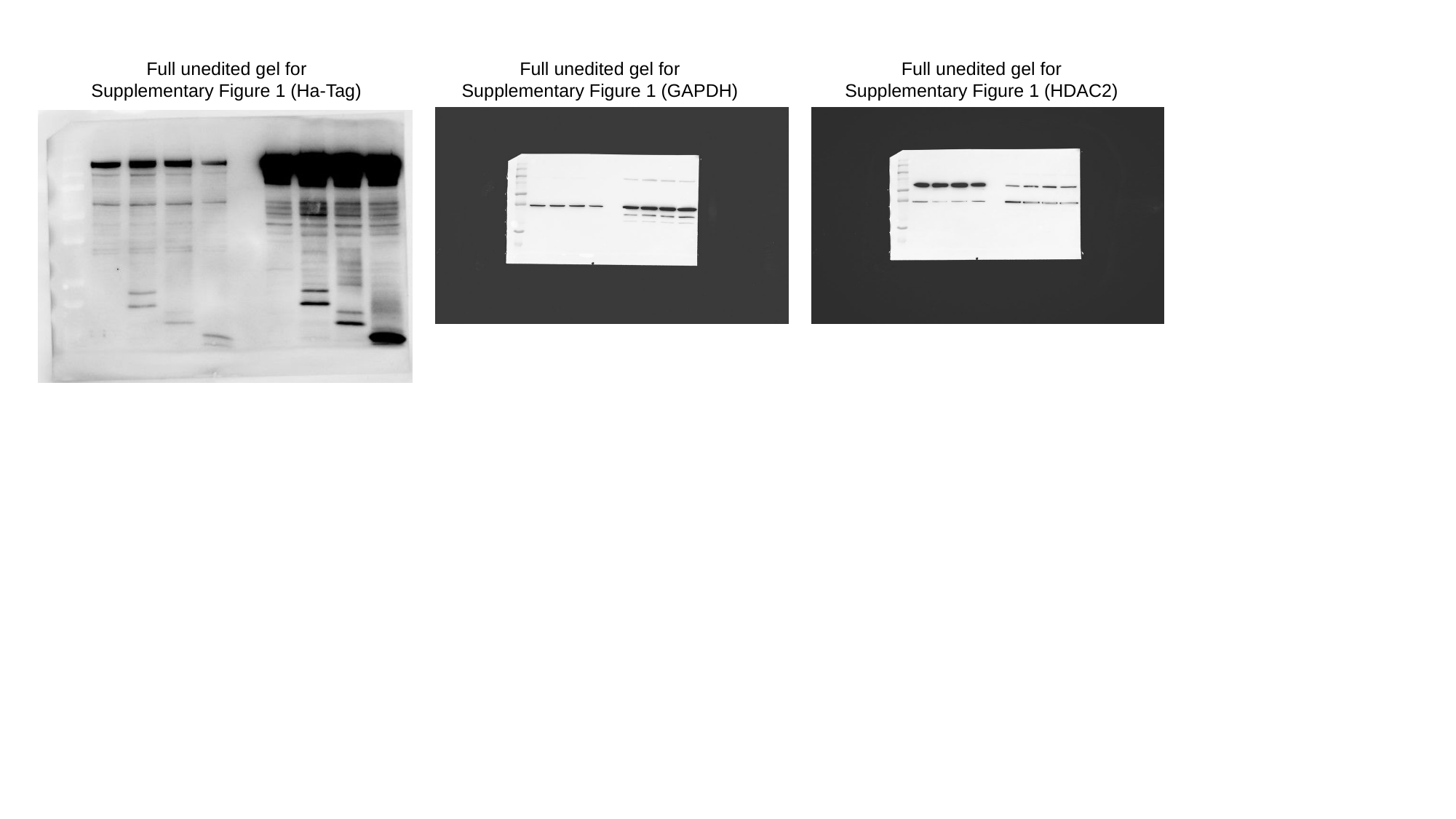

Full unedited gel for
Supplementary Figure 1 (Ha-Tag)
Full unedited gel for
Supplementary Figure 1 (GAPDH)
Full unedited gel for
Supplementary Figure 1 (HDAC2)

## Slide 5
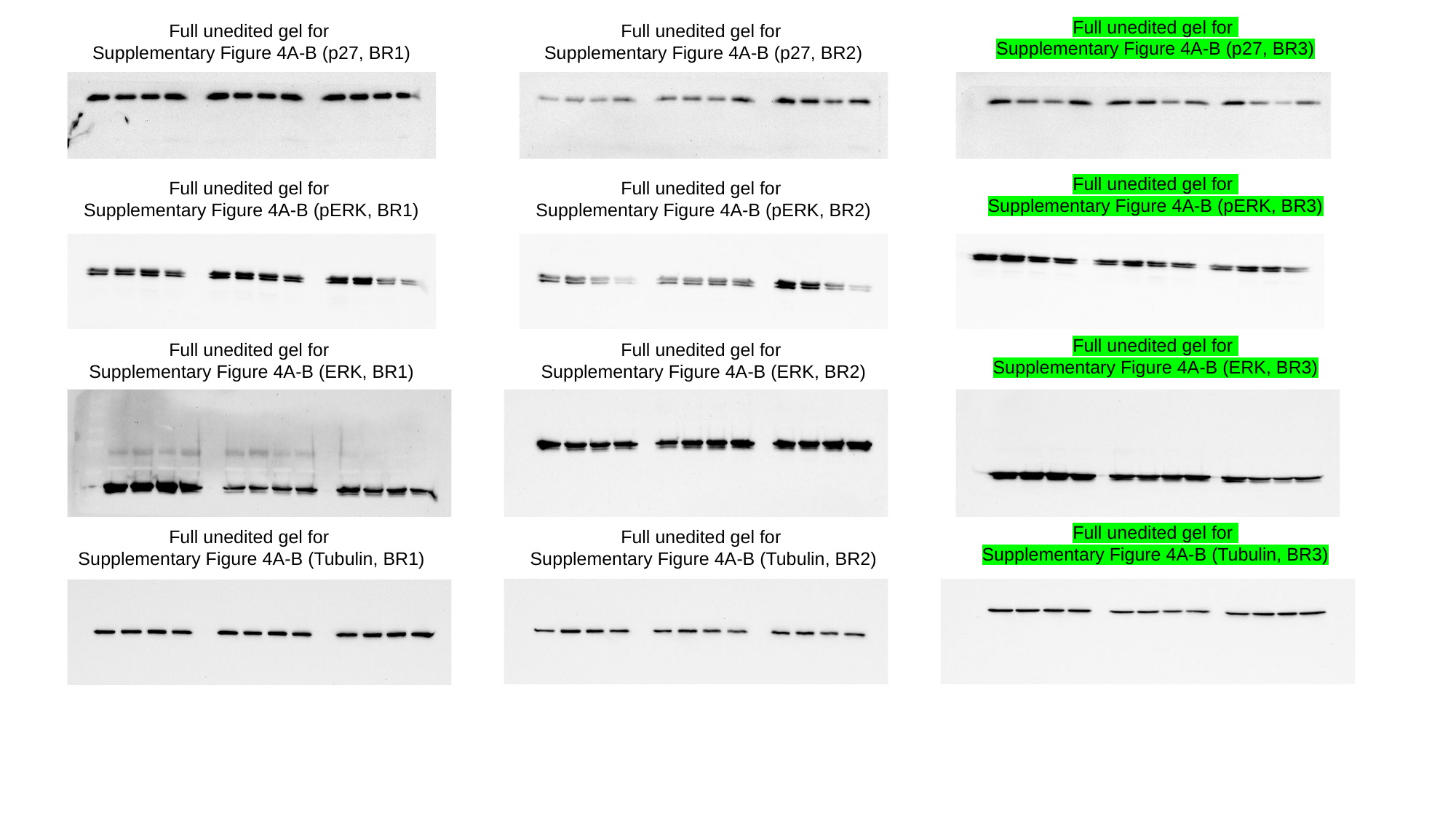

Full unedited gel for
Supplementary Figure 4A-B (p27, BR3)
Full unedited gel for
Supplementary Figure 4A-B (p27, BR1)
Full unedited gel for
Supplementary Figure 4A-B (p27, BR2)
Full unedited gel for
Supplementary Figure 4A-B (pERK, BR3)
Full unedited gel for
Supplementary Figure 4A-B (pERK, BR1)
Full unedited gel for
Supplementary Figure 4A-B (pERK, BR2)
Full unedited gel for
Supplementary Figure 4A-B (ERK, BR3)
Full unedited gel for
Supplementary Figure 4A-B (ERK, BR1)
Full unedited gel for
Supplementary Figure 4A-B (ERK, BR2)
Full unedited gel for
Supplementary Figure 4A-B (Tubulin, BR3)
Full unedited gel for
Supplementary Figure 4A-B (Tubulin, BR1)
Full unedited gel for
Supplementary Figure 4A-B (Tubulin, BR2)

## Slide 6
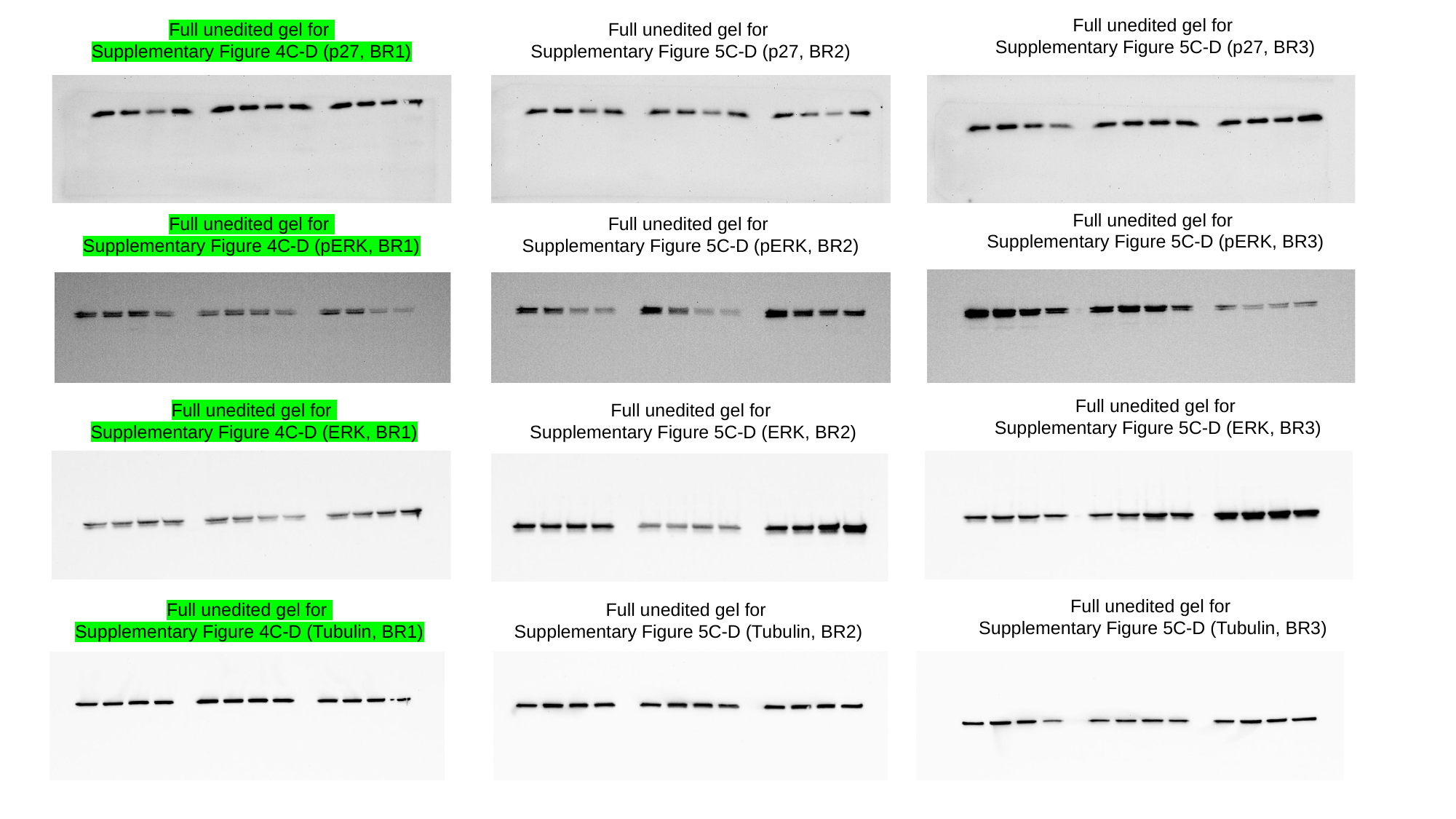

Full unedited gel for
Supplementary Figure 5C-D (p27, BR3)
Full unedited gel for
Supplementary Figure 4C-D (p27, BR1)
Full unedited gel for
Supplementary Figure 5C-D (p27, BR2)
Full unedited gel for
Supplementary Figure 5C-D (pERK, BR3)
Full unedited gel for
Supplementary Figure 4C-D (pERK, BR1)
Full unedited gel for
Supplementary Figure 5C-D (pERK, BR2)
Full unedited gel for
Supplementary Figure 5C-D (ERK, BR3)
Full unedited gel for
Supplementary Figure 4C-D (ERK, BR1)
Full unedited gel for
Supplementary Figure 5C-D (ERK, BR2)
Full unedited gel for
Supplementary Figure 5C-D (Tubulin, BR3)
Full unedited gel for
Supplementary Figure 4C-D (Tubulin, BR1)
Full unedited gel for
Supplementary Figure 5C-D (Tubulin, BR2)
