# Supplementary material for: PTHrP intracrine actions divergently influence breast cancer growth through p27 and LIFR
Source: Breast Cancer Res. 2024 Feb 26;26:34. doi: 10.1186/s13058-024-01791-z (PMC10897994; doi:10.1186/s13058-024-01791-z)
Supplement: Supplementary file 3 — Supplementary Material 3 [file 13058_2024_1791_MOESM3_ESM.docx]

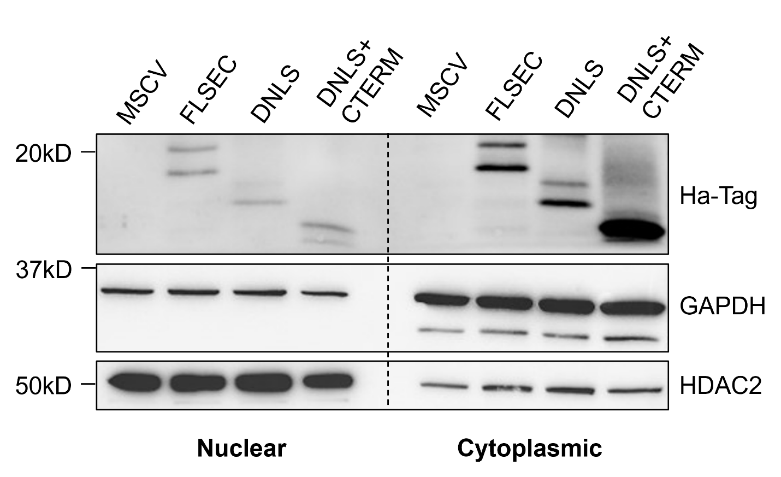


**Supplementary Figure 1.** **Subcellular localization of PTHrP peptides.** (A) Western blot analysis for HA-Tag from nuclear and cytoplasmic fractions obtained from PTHrP mutant cells. GAPDH = cytoplasmic loading control, HDAC2 = nuclear loading control.


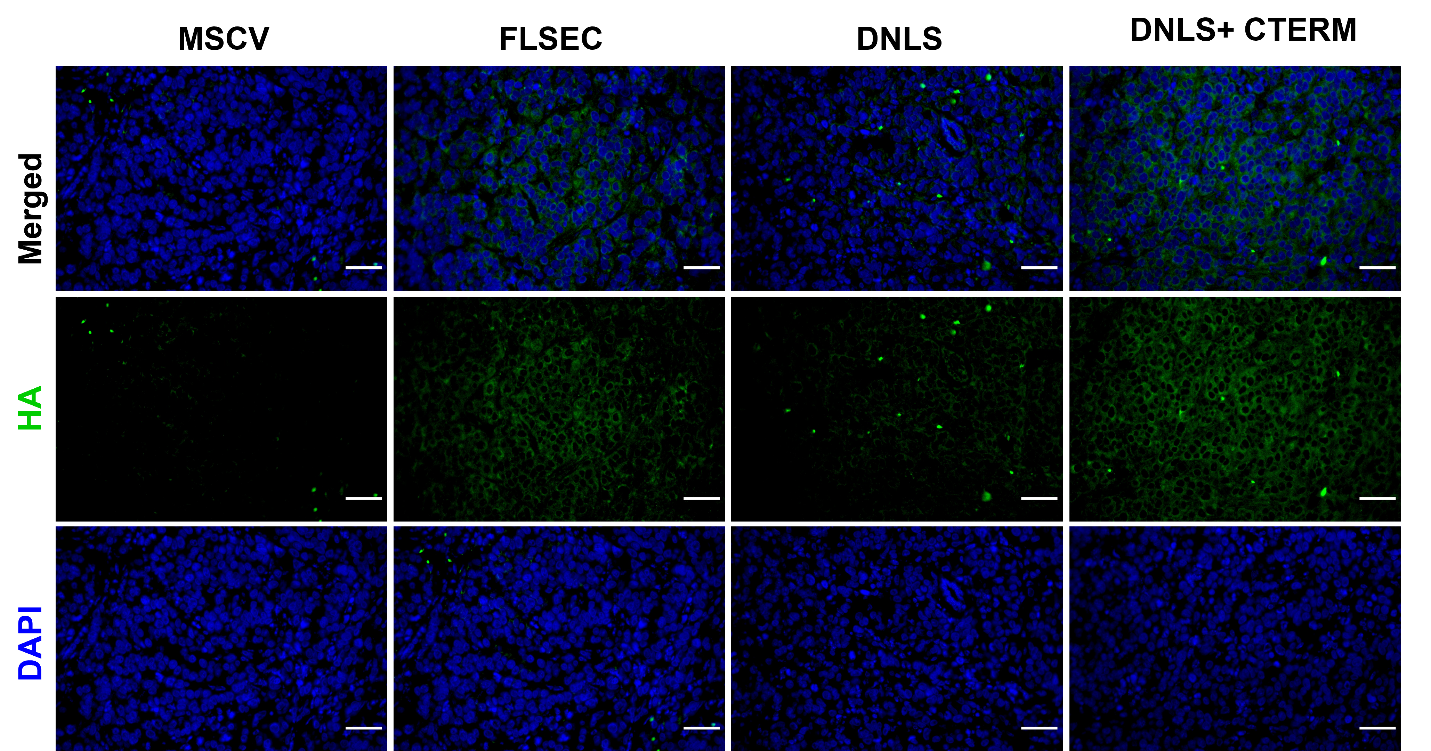


**Supplementary Figure 2. Plasmids expressing specific PTHrP peptides are retained in breast tumors *in vivo*.**  Anti-HA immunofluorescence staining from primary tumors of mice inoculated with MSCV, FLSEC, DNLS, or DNLS+CTERM cells. All panels = 40X and scale bars = 50$\text{μ}$m.


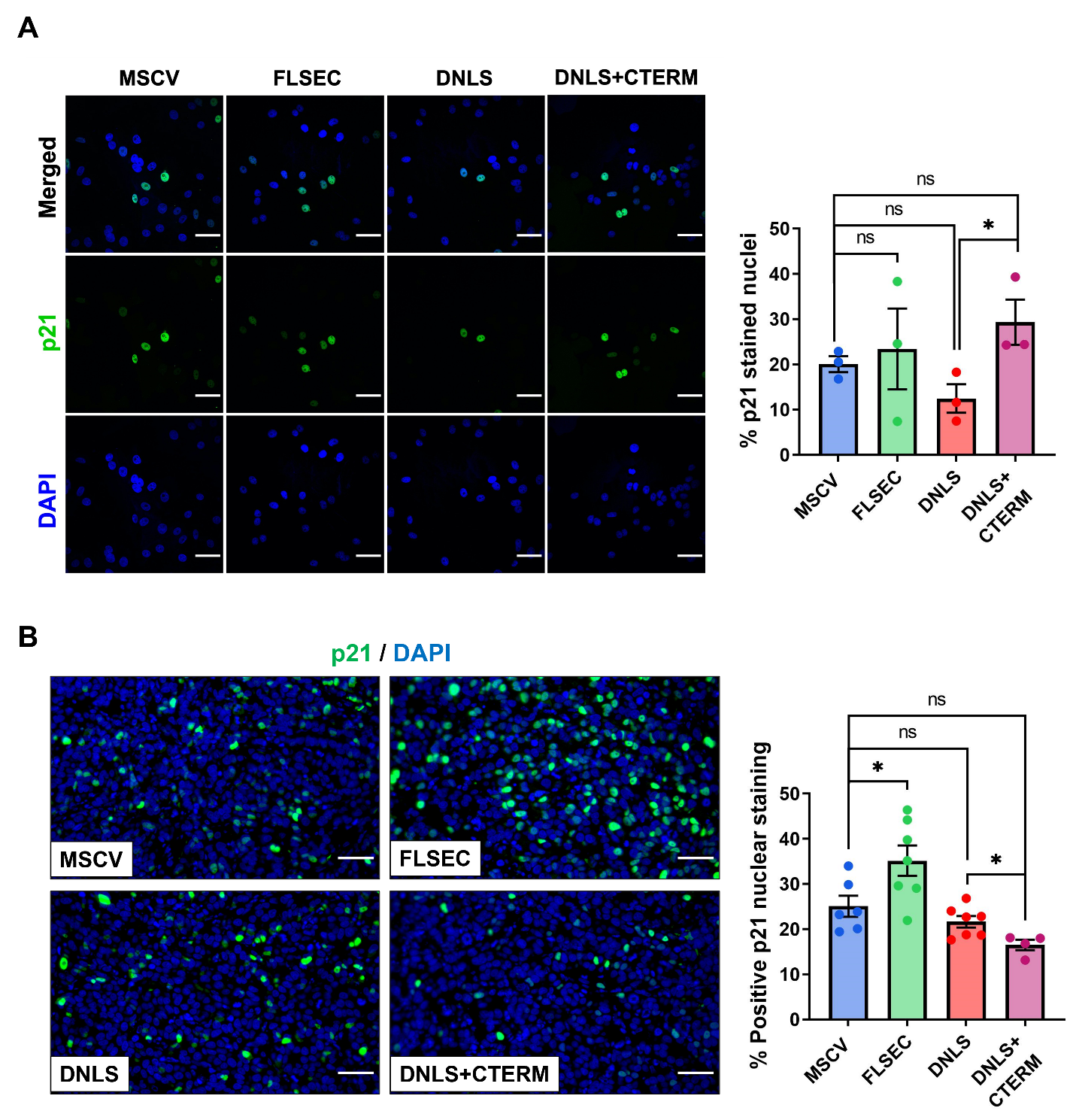


**Supplementary Figure 3**. **Expression of p21 in breast cancer cells expressing different domains of the PTHrP molecule.** (A) Immunocytochemical staining and quantification for p21 in MSCV, FLSEC, DNLS, or DNLS+CTERM cells. N= 3 independent biological replicates. All panels = 40X and scale bars = 25$\text{μ}$m (B) Immunofluorescence staining and quantification for p21 in primary tumors from mice inoculated with MSCV, FLSEC, DNLS, or DNLS+CTERM cells. All panels = 40X and scale bars = 50$\text{μ}$m (A) *p<0.05 vs DNLS by unpaired t-test. (B) *p<0.05 vs MSCV by one-way ANOVA with multiple comparisons or *p<0.05 vs DNLS by unpaired t-test. Graphs represent mean ± SEM.


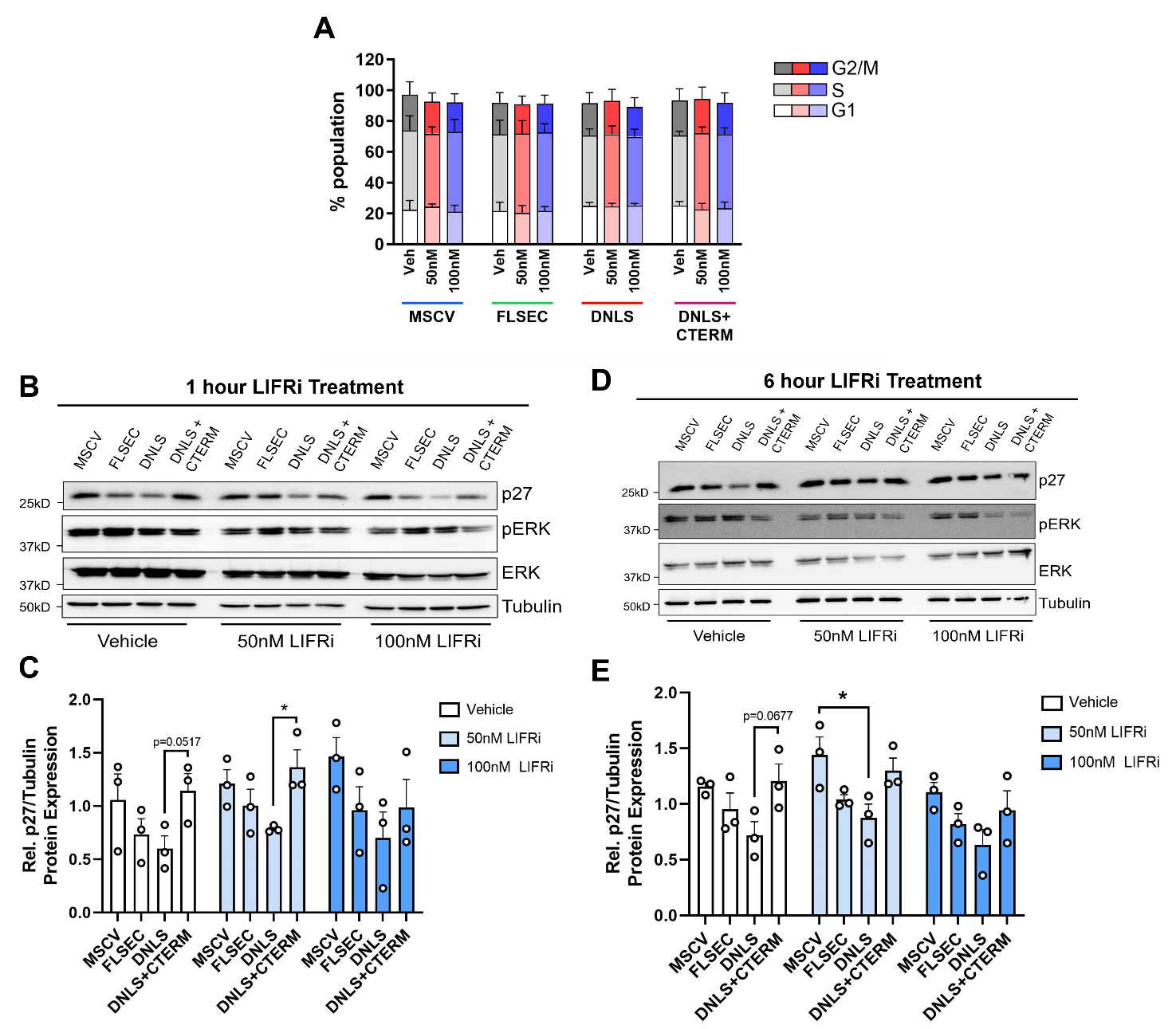


**Supplementary Figure 4**. **Cell cycle and impact of** **short-term LIFR inhibitor treatment on p27 and ERK signaling in PTHrP mutant cells.** (A) Cell cycle analysis of MSCV, FLSEC, DNLS, or DNLS+CTERM cells treated with LIFR inhibitor (EC359, 50nM or 100nM) for 48 hours. (B-D) Western blot analysis and densitometry of p27, pERK, ERK and tubulin (loading control) protein levels in MSCV, FLSEC, DNLS, or DNLS+CTERM cells treated with LIFR inhibitor (EC359, 50nM or 100nM) for 1 hour or 6 hours. (B) *p<0.05 vs DNLS by unpaired t-test. (D) *p<0.05 vs MSCV by one-way ANOVA with multiple comparisons. Graphs represent mean ± SEM.


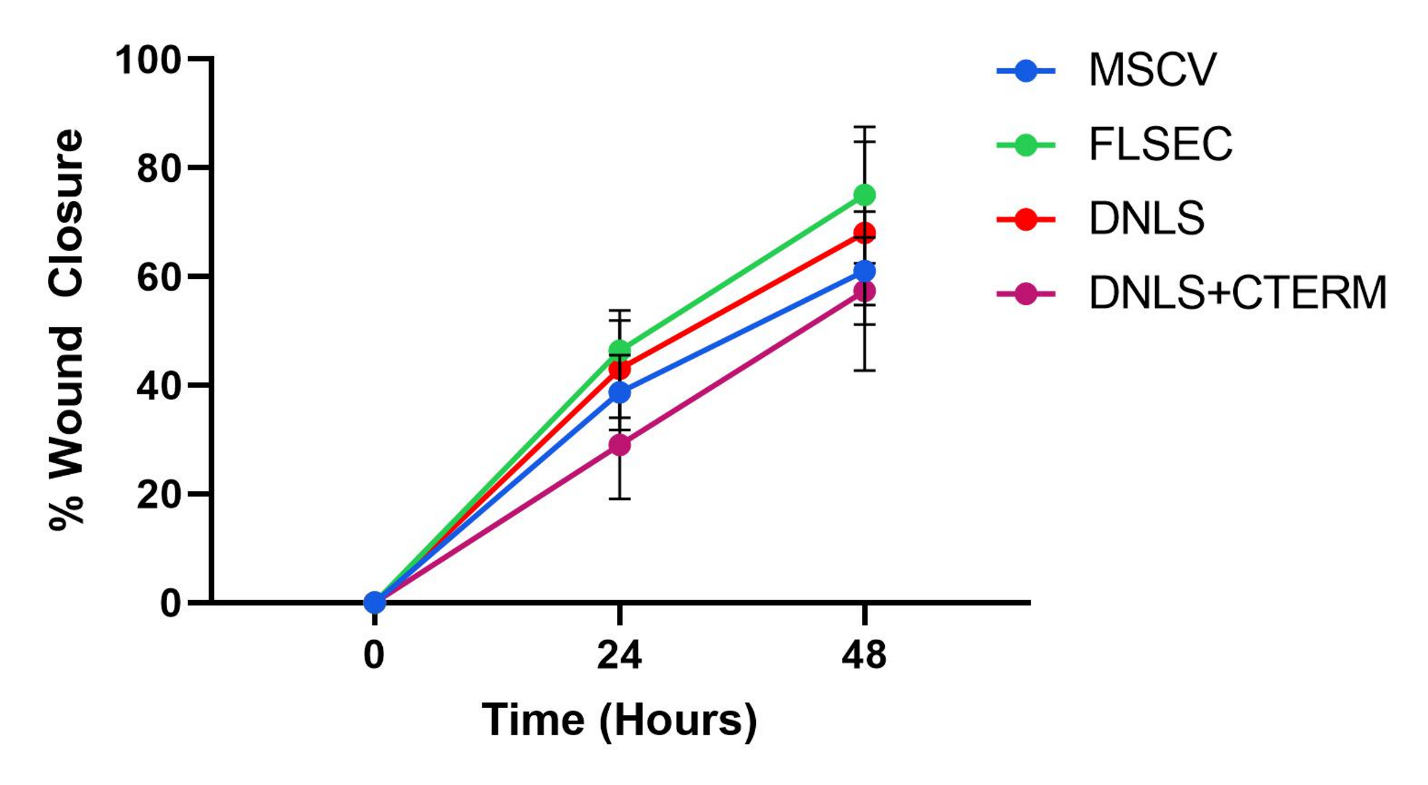


**Supplementary Figure 5**. **Expression of PTHrP fragments does not alter migration.** Scratch assay of MSCV, FLSEC, DNLS, or DNLS+CTERM cells assessed at 24 and 48 hours *in vitro***.** Symbol at each time point represents mean ± SEM of three biological replicates from independent experiments (each biological replicate was averaged from three technical replicates).
